# Investigation on the Keggin Anchored on Hydroxide-Functionalized Single-Walled Carbon Nanotubes as Superior Cathode for Aqueous Zinc-Ion Batteries

**DOI:** 10.1021/acsomega.5c05213

**Published:** 2025-08-05

**Authors:** Langson Chilufya, Vahide Sertbaş, Ahmet Aytekin, Engin Karabudak, Mehtap Emirdag-Eanes

**Affiliations:** Department of Chemistry, Faculty of Science, Izmir Institute of Technology, Gülbahçe Campus 35430 Urla, İzmir 35050, Turkey

## Abstract

Rechargeable aqueous zinc-ion batteries (AZIBs) have become a viable option in electrochemical energy storage systems (EESS) owing to their inherent safety features and economic friendliness. Nonetheless, creating suitable cathode materials for AZIBs with high structural stability, good rate performance, and great capacity remains a significant challenge. Polyoxometalate (POM)-based nanohybrid materials have shown promising results in high cycling stability and great specific capacity. However, POMs susceptible to electrolyte dissolution and the sluggish Zn-ion (Zn^2+^) kinetics have significantly hampered their electrochemical performance as cathodes for AZIBs. Herein, we present a Keggin POM, K_3_[PW_12_O_40_]·*n*H_2_O (KPW_12_), anchored on hydroxyl (OH)-functionalized single-walled carbon nanotubes (SWOH) that were fabricated via a facile ultrasonication procedure. Employed as cathodes for AZIBs, the optimal KPW_12_/SWOH feature exhibited remarkable electrochemical performance. The system satisfied the Zn^2+^ storage, achieving a reversible discharge capacity of 183 mAh g^–1^ at a high current density of 5C with a flat and long discharge plateau after 160 cycles. The perfect synergistic contribution of the pseudocapacitive nature of the super-reduced state of KPW_12_ and the electron-conductive network of SWOH was attributed to this exceptional electrochemical performance. Furthermore, the presence of oxygen in SWOH enhanced the transfer kinetics of electrons and smooth Zn^2+^ diffusion while lowering the Zn^2+^ migration energy barrier by providing more accessible active sites. This demonstrates remarkable promise in fabricating robust electrode materials optimized for integration within aqueous battery systems that pave the way for further research into POM-based materials for EESS.

## Graphical abstract

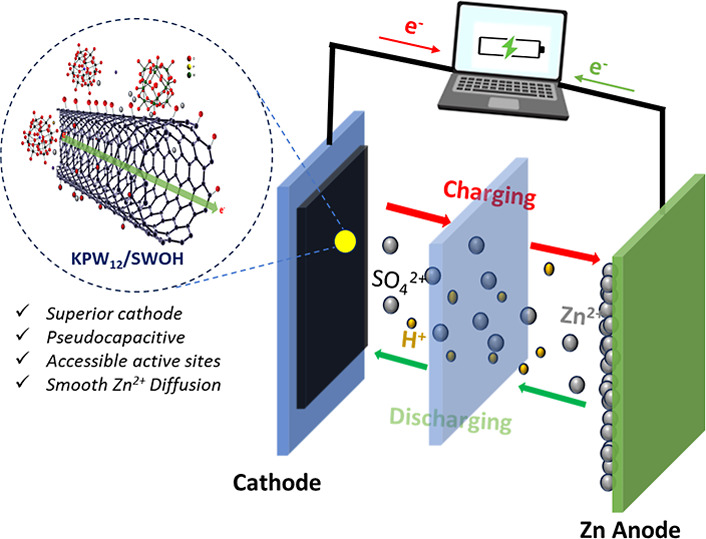

## 1 Introduction

The progression toward green and low-carbon energy generation technologies has been crucial for advancing an all-encompassing clean energy framework and enhancing energy security vital for environmental sustainability.^1^ Nonetheless, green energy generation sources such as wind, hydro, and solar are inherently constrained by seasonal and weather fluctuations and, henceforth, face challenges in becoming reliable and consistent energy providers. Suitable electrochemical energy storage systems (EESS), such as batteries, have been devised to cache the generated electric energy and can additionally realize carbon neutrality with an emission-free automotive sector via electric vehicles (EVs) or as stationary energy battery storage systems for private households and industrial use.^2,3^ Lithium–ion batteries (LIBs), a Nobel Prize-winning technology, have become indispensable energy storage devices in modern society because of their remarkable energy density. They currently lead the EESS technologies employed widely in all applications utilizing rechargeable batteries from portable electronics to EVs, and their extended utilization in large-scale electric grids.^4^ Despite this dominance, LIBs face impediments due to constraints such as finite reserves of lithium ore resources, inherent safety risks associated with flammable organic electrolytes, high costs of operation, and complex manufacturing processes.^5,6^ Consequently, a significant motivation exists for advancing alternative and next-generation EESS, especially multivalent-ion batteries exemplified by Zn^2+^, Mg^2+^, Ca^2+^, and Al^3+^, which are distinguished by their elevated theoretical capacity, ample resource availability, and enhanced safety features.^7^

Among these alternatives, aqueous zinc-ion batteries (AZIBs) with excellent theoretical prospects have been considered promising options, especially in large-scale grid energy applications.^8^ In analogy to LIBs, the fundamental electrochemical processes of AZIBs hinge on Zn^2+^ moving back and forth between Zn metal anodes and the matched cathode.^9^ Therefore, most research has focused on utilizing this Zn metal anode that offers better options in contrast to the lithium metal anode, being high in natural abundance, nontoxic in nature, environmentally friendly, and relatively economical in cost.^10^ Additionally, these Zn-metal anodes have a low redox potential of −0.76 V versus the standard hydrogen electrode (SHE) and highly competitive theoretical capacities of 820 mAh g^–1^ and 5851 mAh cm^–3^. Another vital characteristic is that the Zn anode shows compatibility with both aqueous electrolytes and nonaqueous electrolytes.^11^ Therefore, these AZIB systems are recognized for their excellent inherent attributes, making them ideal candidates for EESS.

Though research has advanced, the practical deployment of AZIBs remains in the early stages of infancy and, unfortunately, has largely been impeded by the inadequate electrochemical performance of cathode materials.^12^ Creating suitable cathode materials that offer high structural stability, good rate performance, and high capacity continues to be a significant challenge.^13^ Numerous cathode materials have been proposed and reported in literature, including Mn- or V-based oxides, Prussian blue derivatives, layered sulfides, and organic materials.^14^ Unfortunately, the cycling stability of these cathodes often suffers due to volume expansion and crystalline lattice changes during Zn-ion intercalation/deintercalation cycles, leading to low capacity, severe battery polarization, and rapid capacity degradation.^15,16^

Recently, polyoxometalate (POM)-based compounds have been advanced as cathodes for secondary batteries such as AZIBs, giving promising results and impressive electrochemical performance.^17^ POMs are a fascinating class of inorganic polynuclear anionic frameworks comprising high-valent transition metals, typically based on V, Mo, W, and so on bonded to oxygen. Some of these metal oxide heterometals from throughout the periodic table can be introduced into these frameworks.^18^ They are crystalline structures that exhibit unique redox properties, which have led to extensive application in catalysis, sensors, and energy conversion/storage.^19–23^ Generally, in EESS, they can effectively incorporate redox-active groups into the POM framework with no significant structural changes that make them stand out from other compounds. This allows for reversible multielectron accepting/donating and high storage capacity, whereby the concept of the “electron/ion sponge” phenomenon is articulated to elucidate such behaviors effectively.^24–28^ The unrivalled structural characteristics, like high intrinsic electrons, high ion storage capacity, and chemical diversity in tunability, have made POMs emerge as a singularly intriguing candidate for use as cathode materials in AZIBs. For instance, Park et al.^29^ elucidated that cationic exchange on {PMoV} may extend the operational voltage range, enhance the charge storage kinetics and reversibility of {HPMoV}, and promote the intercalation pseudocapacitance mechanism. The {KPMoV} cathode exhibited an exceptional continuous capacity of 74.0 mAh g^–1^ at a current density of 20 A g^–1^ and demonstrated an extended cycling lifespan exceeding 2000 cycles at a current density of 1 A g^–1^, thereby outpacing other molybdenum-based cathode materials. Zhou et al.^30^ demonstrated a polyoxovanadate-type K_4_Na_2_V_10_O_28_ (KNVO) capable of delivering a specific capacity of 152.3 mAh g^–1^ at a current density of 0.1 A g^–1^ for over 100 cycles. Furthermore, KNVO exhibited an outstanding electrochemical performance at 0.1 A g^–1^, with almost 100% of the capacity retained at a high current density of 1 A g^–1^ after 1000 cycles.

However, the POMs’ inherently negligible electronic conductivity, prone to dissolve in electrolyte, and strong oxygen interaction with electroactive materials impede reversible process of ions and easy diffusion, leading to lower reversible capacity and suboptimal cycling performance, respectively. Interestingly, researchers have advanced POM-based materials with innovative structural design and open channels that facilitate rapid ion transport and stable intercalation to address these challenges in EESS.^31,32^ Generally, extensive strategies have been developed to address these challenges, which include immobilizing POM on conductive substrates like carbonaceous materials in most EESS.^33,34^ Particularly, in AZIBs, where most cathodes are less conductive and undergo volume changes, these carbon materials overcome these by enhancing the electric conductivity, mitigating the volume alteration, shortening ion transport, and electrolyte diffusion distance.^35^ Carbon nanotubes (CNTs) with a 1-D tubular structure have been proposed as ideal conductive substrates for the electroactive cathode materials in AZIBs. Their conductive network boosts the Zn^2+^ storage by enhancing the rapid diffusion of Zn^2+^ and the tight contact between the electrode and electrolyte.^36^ Therefore, they continue to draw interest due to their outstanding electrical conductivity, large surface area, and strong electrochemical stability.^37–40^ For example, Lui et al.^41^ reported an enhanced electrochemical performance of α-MnO_2_ nanofibers/CNT hybrid as a high-capacity cathode material for AZIBs than the pristine α-MnO_2_ nanofibers. The nanohybrid delivered an impressive specific capacity of around 296 mAh g^–1^, maintaining 96% of its capacity at a current density of 0.2 mA g^–1^ over more than 100 cycles. Fan et al.^42^ were able to demonstrate on a β-VO_2_/CNTs core–shelled microspheres, that displayed an outstanding specific capacity of 380 mAh g^–1^ at the current density of 0.05 A g^–1^, with 80% capacity retained for over 7800 cycles. It can be deduced that due to the synergistic effects, the grafting of these metal oxide materials onto CNT, their structural integrity, electronic conductivity, and chemical stability of the resulting nanohybrid are significantly improved during Zn^2+^ intercalation/deintercalation.

Functionalizing CNT has been regarded as one of the best approaches in improving both the physical and chemical properties of CNT. For instance, the oxygen-functionalized CNTs have surface functional groups that can act both as anchoring sites of the electroactive material and suppress the aggregation of POMs.^43^ More interestingly, the oxygen on the surface of CNT can either facilitate electron transfer between metals and carbon atoms or engage in direct interaction with metal, thereby serving a pivotal function as an adhesive agent between the metal and the CNT surfaces.^44^ Additionally, they can enhance the electrolyte’s wettability and the surface reactivity of the cathodes even with the less amounts of active materials.^45,46^ Therefore, this can facilitate the adsorption and desorption of electroactive ions during the redox process, resulting in enhanced electrochemical performance of the EESS. According to the authors’ knowledge, none of the POMs have been anchored on CNT for cathode application in AZIBs. Henceforth, it is worth attempting to design and fabricate high-performance POM/CNT nanohybrid cathodes for AZIBs, which present extensive potential to exploit synergistic interactions among constituent materials.

In the scope of this study, the need for a highly stable POM as a prospective cathode active material in AZIBs with an enhanced electrochemical performance is imperative. We systematically investigated energy storage mechanisms for a series of K_3_[PW_12_O_40_]·*n*H_2_O (KPW_12_) and its nanohybrids, K_3_[PW_12_O_40_]/SWCNT (KPW_12_/SW) and K_3_[PW_12_O_40_]/SWCNT-OH (KPW_12_/SWOH), that were designed via a simple ultrasonication technique. Incorporating a hydroxide-functionalized CNT significantly inhibited the electrolyte dissolution and structural degradation of KPW_12_, thus augmenting the enhanced electrochemical activity of this cathode. Consequently, the as-prepared KPW_12_/SWOH exhibited significantly enhanced zinc storage performance with exceptional rate capability, delivering a higher specific capacity of 183 mAh g^–1^ at a high current density of 5C, with 94% capacity retained, coupled with a good cycling stability of minimal capacity decay that was observed after 160 cycles. Encouragingly, this nanohybrid displayed a superior Zn^2+^ storage performance, with an outstanding reversible discharge capacity of 350.8 mAh g^–1^ after 50 cycles at a higher current density of 10C. This was ascribed to the microchannel tubular CNT structure that acted as a tunnel for electron transport, improved the intrinsic conductivity, and enhanced the capacity retention and cycling stability of redo-active KPW_12_ cathode materials. Additionally, employing functionalized SWCNT-OH created oxygen vacancies, which enhanced the reactive sites and facilitated the Zn^2+^ diffusion kinetics. This research provides a straightforward and effective strategy to advance the use of POM-based cathodes in AZIBs.

## 2 Results and Discussion

### 2.1 Materials Synthesis and Characterization

The synthesis of the nanohybrids was carried out as demonstrated in Scheme 1, following the literature methods.^47,48^ First, the bundled CNTs were dispensed by ultrasonication, while the suspension formed by the reaction mixture of KCl and H_3_PW_12_O_40_·*n*H_2_O was hydrothermally prepared at 150 °C for 12 h. Subsequently, the KPW_12_ formed underwent nanohybridization with SW or single-walled carbon nanotubes (SWOH) via a facile ultrasonication process. The methodology section of the Supporting Information outlines a detailed description of the procedure.

**Scheme 1:**
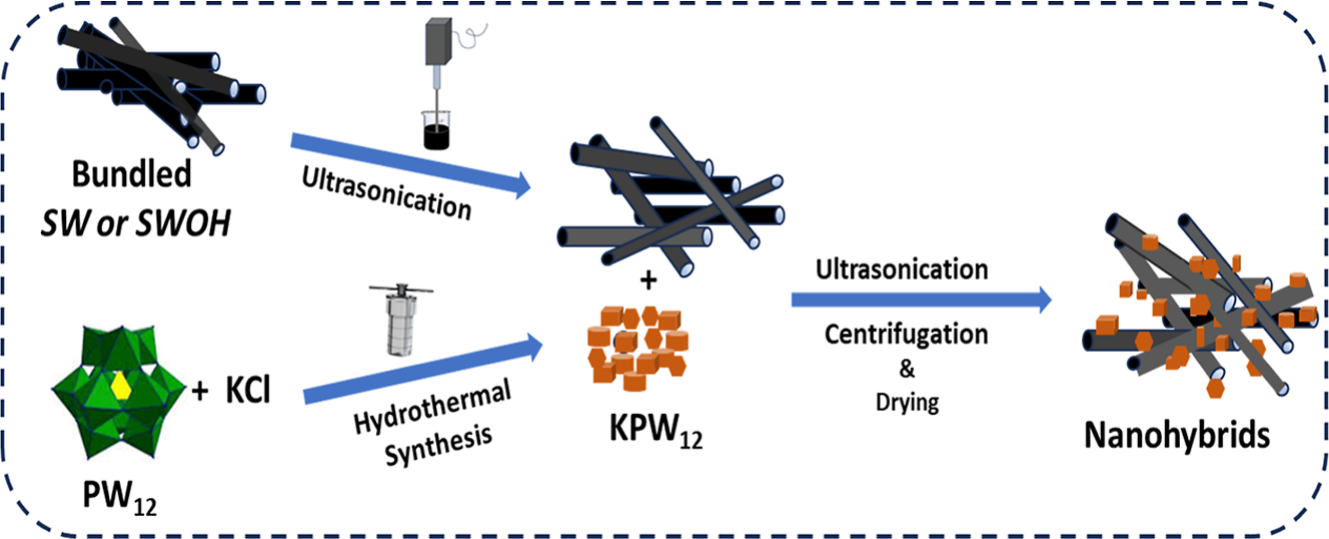
Preparation of the Nanohybrids

In the examination of morphology, scanning electron microscopy (SEM) was employed, and as shown in Figure 1a, the KPW_12_ microstructures with a relatively smooth surface at 5 μm. The morphology of the SW and SWOH, as given in Figure S1a,b, respectively, shows a tubular-like chain structure consistent with the CNT structure. After the addition of the SWOH, the as-prepared KPW_12_/SWOH nanohybrid shows a well-defined nanorod morphology structure like a chain without aggregation, as shown in Figure 1b, and the same can be said for KPW_12_/SW nanohybrid (Figure S1c) at 500 μm.^49^ Additionally, a relatively smooth surface of SWOH with some wrinkles of the stacked KPW_12_ can be observed, suggesting good crystalline and good POM “wiring” on the CNT of the as-prepared nanohybrids.^50^ The elemental analysis was done with an energy dispersive X-ray (EDX) spectrometer, showing the percentage of KPW_12_, excluding the carbons from SWOH, is demonstrated in Figure 1c, which was consistent with the typical distribution of elements in KPW_12_. The corresponding EDX mapping is shown in Figure 1d, revealing the homogeneous distributions of W, P, and O of POM anions onto the CNT substrate, suggesting the successful immobilization.^51^

**Figure 1:**
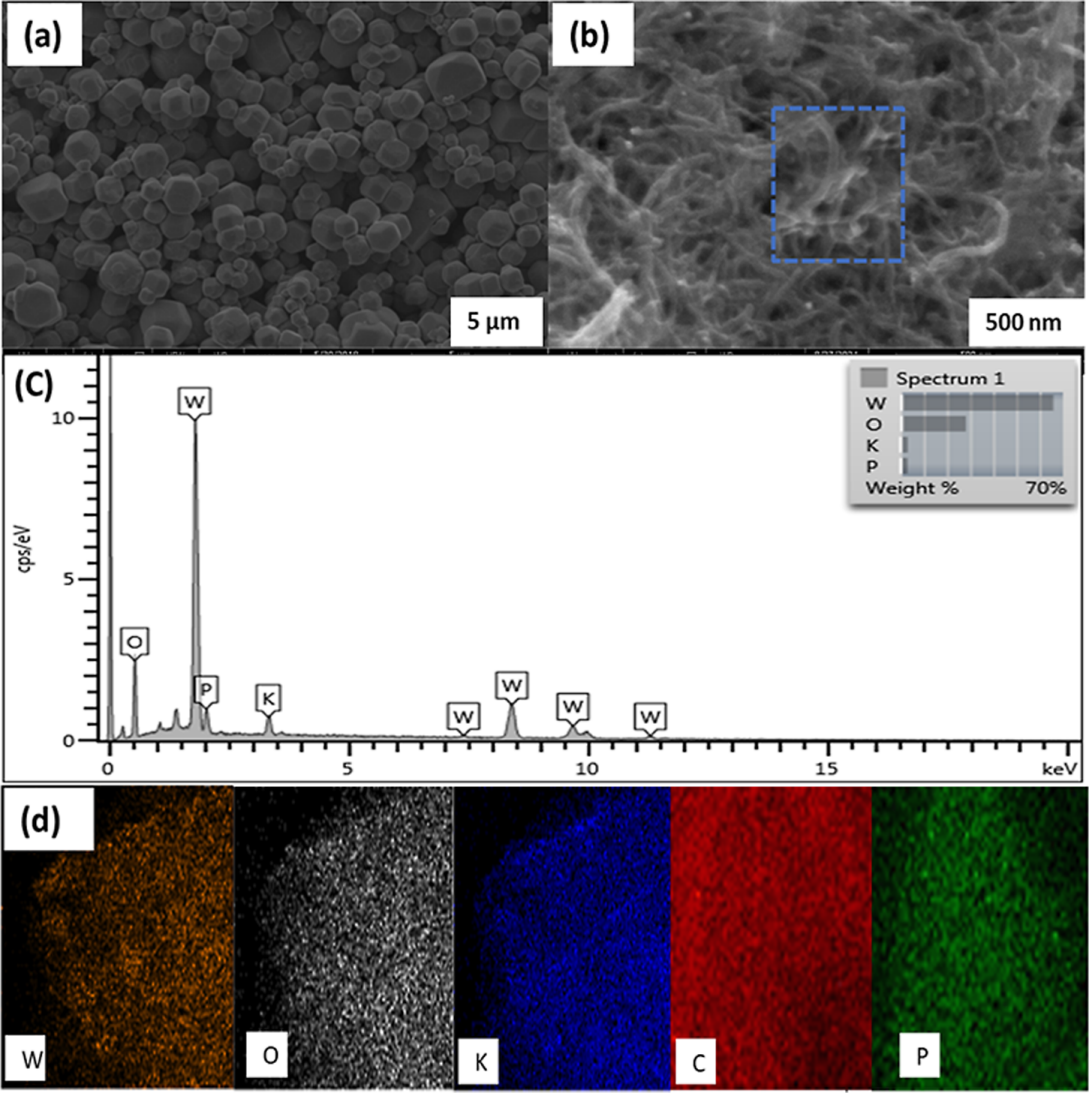
(a)­The SEM images of KPW_12_ done at 5 μm. (b) The SEM images of KPW_12_/SWOH at 500 nm. (c) The EDX spectra of KPW_12_/SWOH. (d) The EDX elemental mapping images of KPW_12_/SWOH.

The functional groups, structural, and bonding changes present in the KPW_12_, KPW_12_/SW, and KPW_12_/SWOH nanohybrids were predicted using the FTIR spectrometer. The spectra were recorded by blending the samples with KBr pellets and compressing them into plates for testing, within the wavenumber range of 450–4000 cm^–1^. As shown in Figure 2a, KPW_12_ has four typical peaks that are characteristic of Keggin POM.^52,53^ The characteristic stretching vibrations were obtained with bands associated with the ν_as_(P–O) vibrations around 1078 cm^–1^, the terminal ν_as_(W–O_t_) occurs at 978 cm^–1^, the corner-sharing ν_as_(W–O_b_–W) observed at 889 cm^–1^, and edge-sharing ν_as_(W–O_c_–W) vibrational modes present at 811 cm^–1^. These signature peaks of KPW_12_ clusters were spotted in the two nanohybrids’ spectra, confirming the successful anchoring of the KPW_12_ clusters onto SW and SWOH. However, the spectrum showed weak intensity of most of the bands, likely due to the small quantity of KPW_12_ clusters used in fabricating these nanohybrids. Moreover, the intensity of the ν_as_(P–O) stretching bands shifted slightly toward higher wavenumbers as observed in the nanohybrids, suggesting an electronic interaction between KPW_12_ and SW/SWOH. The ν_as_(O–H) broad absorption band from water molecules was clearly observed starting at 3400 cm^–1^, indicating their presence in the POM. It was even more pronounced with high intensity in the KPW_12_/SWOH nanohybrid.^50^ Furthermore, the typical bonds of CNT backbone are present in the nanohybrids with the peak value around 2490 cm^–1^, indicative of C–O stretch for the SWOH and C=C skeletal vibration of SW.^54^

**Figure 2:**
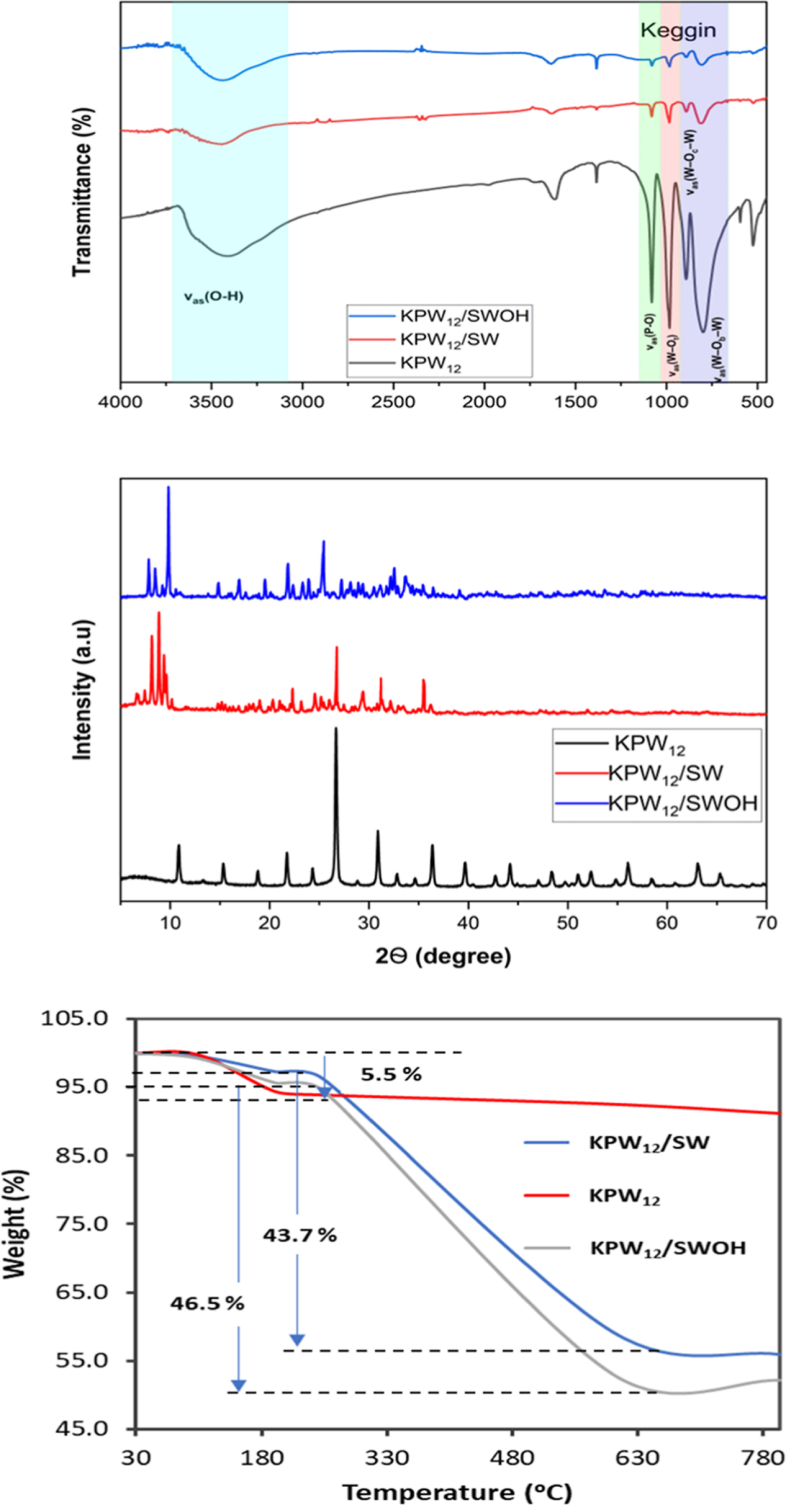
(a) XRD spectrum showing the characteristic peaks. (b) The FT-IR spectrum shows the Keggin and SWCNT characteristic vibrations in the nanohybrid. (c) The TGA curves showing the weight loss of the samples.

Powder XRD was conducted to determine the crystalline phases of the samples. The XRD patterns (Figure S2) of KPW_12_ crystalline structure show all characteristic peaks at 2θ value of approximately 10.8°, 26.7°, 31.4°, and 36.6° that match well with PW_12_ (JCPDF No. 06-0518) without any phase impurities corresponding to the (002), (642), (102), and (222).^48,55,56^ The XRD spectra of the two nanohybrids shown in Figure 2b, the distinctive peaks can be assigned to high crystallinity, indicating that the samples are both well-prepared with no phase impurities, though they were covered in CNT. However, due to the amorphous nature of the CNT in the XRD patterns (Figure S3), sharp peaks can be observed for KPW_12_ clusters, which reflects some good crystallinity of the obtained nanohybrid material.^57^ This suggests considerable anchoring of KPW12 crystals on the surface of CNT, consistent with the SEM results. These findings validate the effective synthesis of the POM/CNT samples, with no significant alterations observed in their crystal structures following ultrasonication treatment. The thermostability of these nanohybrids’ crystalline material was examined via thermogravimetric analysis (TGA) from a temperature range of 10–800 °C, as shown in Figure 2c. An initial average weight loss of 5.5% in the TGA curve in the range 108–196 °C was attributed to the mass loss due to evaporation of physically adsorbed moisture or the elimination of water crystals in the samples.^53^ Constant weight loss of 43.7% and 46.5% for KPW_12_/SW and KPW_12_/SWOH nanohybrids, respectively, is observed in the temperature range of 250–600 °C, resulting from the combustion of the CNT backbone and elimination of the OH functional groups to form CO or CO_2_.^52^ Consequently, weight stabilization was followed by about 50% of KPW_12_ remaining in the nanohybrids. The TGA results further confirmed the formation of POM/CNT nanohybrids, which aligned well with the theoretical predictions and experimental elemental analysis obtained.

### 2.2 Electrochemical Performance

The electrochemical testing apparatus was set, as detailed in the Supporting Information, with 5 M ZnSO_4_ as an electrolyte. To shed light on the outstanding redox properties of the cathodes, cyclic voltammetry (CV) curves at scan rates of 5 mV s^–1^ were analyzed. Figure 3a depicts a CV curve of KPW_12_/SWOH in the voltage range of 0.2–1.8 V after three cycles. The first cycle, which includes an activation process, deviates from the subsequent cycles. In contrast, the second and third cycles exhibit excellent overlap, demonstrating the excellent electrochemical reversibility involving the multistep insertion/deinsertion processes of Zn^2+^ through the KPW_12_ framework.^58^ The oxidation peaks are identified at 0.64, 1.03, and 1.60 V, whereas the corresponding reduction peaks are found at 0.60, 0.81, and 1.36 V. The peaks at 0.46/0.60 V are ascribed to the intercalation of Zn^2+^ into the KPW_12_ framework. The redox couples of W^4+^/W^5+^ and W^5+^/W^6+^ are denoted by the peak pairs at 0.64/0.60 V and 1.60/1.36 V, respectively. Moreover, a reduction peak at 0.81 V is hypothesized to arise from the insertion of H^+^/H_2_O into the KPW_12_ framework, which aligns with prior findings documented in the literature.^46,59^ The CV curve KPW_12_/SW cathodes (Figure S4a) exhibited a shape similar to the KPW_12_/SWOH curve, implying that they exhibit identical electrochemical redox activity. However, the CV curve of KPW_12_/SWOH cathode displayed the most prominent peak current and enclosed area, suggesting the reason it had the highest specific capacity, as we shall demonstrate in this article.^60^ Notably, the CV curves of SW and SWOH, as displayed in Figure S4b,c, did not manifest the obvious redox peaks, indicating they never participated in the redox process.

**Figure 3:**
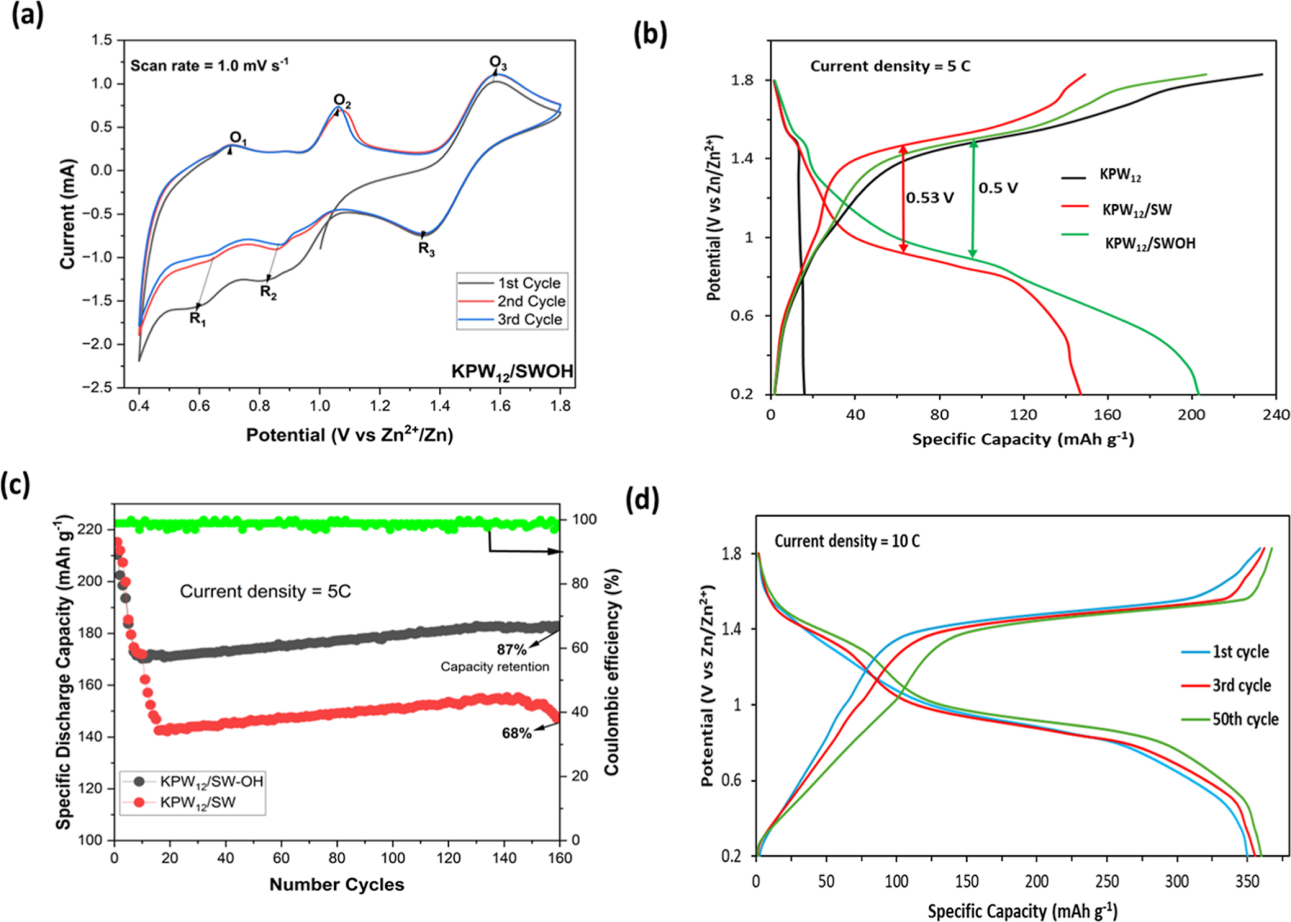
(a) The CV spectrum showing the redox properties of the KPW_12_/SWCNT cathode. (b) The GCD curves for the cathode samples at the current density of 2.0 A g^–1^. (c) The cycling stability of the KPW_12_/SW and KPW_12_/SWOH cathodes at a current density of 5C. (d) The GCD curve of KPW_12_/SWOH at current density of 10C.

The Galvanostatic charge/discharge (GCD) analysis, as illustrated in Figure 3b, presented two different voltage plateaus near 1.05 and 1.65 V that corresponded to the redox process of redox couples of W^4+^/W^5+^ and W^5+^/W^6^, consistent with the CV curves. The sharp rise in the curve indicates the surface redox process of the cathode with high plateaus disclosing the enhanced specific capacity due to nanohybridization of the POM with CNT.^61^ Therefore, the superior cathode electrochemical performance of KPW_12_/SW and KPW_12_/SWOH for Zn-ion storage capacity was obtained with a specific discharge capacity of 147 and 203 mAh g^–1^, respectively, at a current density of 5C (1C = 12.5 A g^–1^). Additionally, the charge–discharge plateaus’ potential gap at 50% state of charge (SOC) was lower for KPW_12_/SWOH (0.5 V) than for KPW_12_/SW (0.53 V), which suggested lower polarization and improved reaction kinetics of the Zn^2+^ in the cathode attributed to its anchoring to SWOH.^62^ Meanwhile, despite showing a higher charge capacity, it was observed that KPW_12_ displayed a poor discharge capacity of 16 mAh g^–1^, which can be attributed to the instability caused by dissolution into the aqueous electrolyte or an irreversible phase transition of POMs. This was proved by examining the behavior of the electrolytes after five cycles with the three cathodes in UV–visible spectroscopy. After repeated cycling, the electrolytes from KPW_12_/SW and KPW_12_/SWOH cathodes exhibited no significant absorption peaks that were observed in the UV–visible spectra (Figure S5), unlike a strong intensity absorption peak that appeared for KPW_12_. The nanohybrids’ electrolytes remained transparent, which indicated excellent structural electrochemical stability, in contrast with the yellow turned electrolyte for KPW_12_, which underwent side reactions, negatively impacting its performance.

The cycling performance was assessed with a current density of 5C, as illustrated in Figure 3c. KPW_12_/SWOH cathode exhibited an impressive discharge capacity of 183 mAh g^–1^ for over 160 cycles with 87% capacity retention in comparison to KPW_12_/SW, which delivered a capacity of only 147 mAh g^–1^ with 68% of capacity retained. Both nanohybrids exhibited high Coulombic efficiencies of almost 99% during charging/discharging cycles. It is worth noting that the KPW_12_/SWOH cathode displayed a better electrochemical performance, which is attributable to the additional active sites from functionalized SWOH that enhanced its specific capacity. Moreover, even at a high current capacity of 10C, KPW_12_/SWOH delivered a specific capacity of 360.8 mAh g^–1^ after 50 cycles, as demonstrated by the GCD curve in Figure 3d, unlike the KPW_12_/SW that gave 214 mAh g^–1^ (Figure S6a).

The rate performance study was obtained at different current densities, as illustrated in Figure 4a. Remarkably, for the KPW_12_/SWOH battery system tested at various current densities from 0.1, 1, 2, 4, to 5C, the cathode can reach average specific discharge capacities of 194, 192, 187, 180, and 175 mAh g^–1^, respectively. This performance was higher than the KPW_12_/SW obtained average specific discharge capacities of 174, 171, 165, 153, and 144 mAh g^–1^, respectively. When the battery is cycled again at 0.1C, the reversible capacity is restored to 193 mAh g^–1^, equivalent to 99.5% of the initial value for KPW_12_/SWOH, while 172 mAh g^–1^ was retained, corresponding to 98% of the initial capacity of KPW_12_/SW. The corresponding GCD curves for the rate performance are shown in Figures 4b and S6b for KPW_12_/SWOH and KPW_12_/SW cathodes, respectively. These findings highlight the excellent reversibility and the strong stability of these nanohybrid crystalline structures. Additionally, the presence of the CNTs not only provided an effective conductive electronic network but also suppressed the structural volume changes during Zn^2+^ intercalation/deintercalation, leading to a stable cathode.^49^

**Figure 4:**
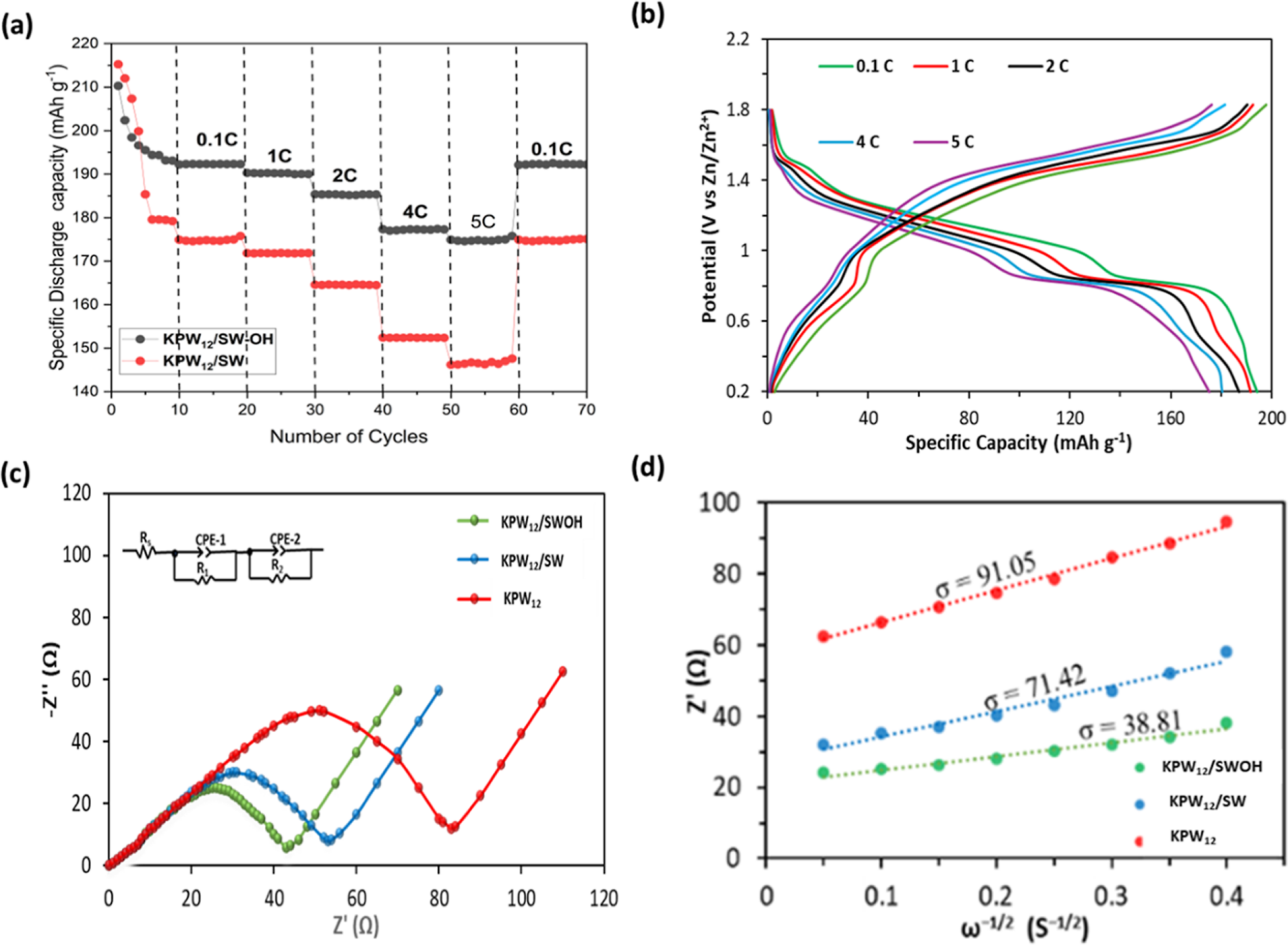
(a) The rate capability of the KPW_12_/SW and KPW_12_/SWOH cathodes at different discharge capacities and current densities. (b) The GCD curve of KPW_12_/SWOH cathodes at various current densities. (c) The typical Nyquist plots of all samples. (d) Fitting curves of *Z*′ and ω^–1/2^ for determination of Warburg coefficient (σ, slope).

The charge transfer and diffusion of Zn^2+^ (D_Zn_^^2+^^) kinetics of these cathodes were analyzed using electrochemical impedance spectroscopy (EIS). Generally, EIS curves with a semicircle in the high- to medium frequency region indicate the charge transfer resistance (*R*_ct_). Meanwhile, the Warburg impedance (*W*), which has an inverse proportional relationship with the ion’s diffusion kinetics within the electrode, is represented by the slash line in the lower frequency region.^63^ The EIS analysis after 50 cycles, as demonstrated in Figure 4c, was evident from the Nyquist plots of all samples that the *R*_ct_ for KPW_12_/SWOH (42.54 Ω) and that of KPW_12_/SW (53.14 Ω) were lower than that of KPW_12_ (85.05 Ω), indicating low resistance in the nanohybrids. This suggested that a fast Zn^2+^ diffusion process was enhanced in nanohybrids due to reduced KPW_12_ cluster agglomeration attributed to the surface anchoring on conductive SW or SWOH, which boosts the transfer of Zn^2+^. This was consistent with the D_Zn_^2+^ kinetics estimated from Warburg coefficient (σ, slope of Z′ vs ω^–1/2^) calculation displayed in Figure 4d and Table S1. The diffusion coefficient, σ values for KPW_12_/SWOH (38.81 Ω ω^–1/2^) exhibited a lower *W*, further indicating a higher D_Zn_^^2+^^ kinetics than KPW_12_/SW (71.42 Ω ω^–1/2^) and KPW_12_ (91.05 Ω ω^–1/2^). This impressive Zn^2+^ diffusion behavior and charge transfer observed in KPW_12_/SWOH nanohybrid should benefit from the synergistic effect of anchoring of conductive SWOH, accompanied by more active oxygen sites availability, which resulted in excellent rate performance. The above conclusion indicates that the multiredox behavior of the POM, PW_12_, is greatly enhanced by the conductive SW and SWOH when employed as cathodes for AZIBs.

In comparison with the electrochemical performance of the previously reported POM-based cathode materials for AZIBs, as shown in Table 1, our product can significantly compete and surpass most of them.^29,30,58,64–67^ Notably, most of these compounds reported in literature involve the use of polyoxovanadates as cathodes that have irreversible byproducts arising from H^+^ insertion and the inherent instability of V-based oxides.^68^ The electrochemical performance was also compared to the non-POM cathode systems, such as organic, MOF-based, and hybrid composites from recently reported literature, where our compound performed fairly well (Table S2).

**Table 1:** Electrochemical Performance of POM-Based Cathodes for AZIBs

cathode materials	electrochemical performance (capacity retention and cycle numbers)	ref.
KPMoV	94.1 mAh g^–1^ at 10 A g^–1^ (98.2% after 400 cycles)	^29^
K_4_Na_2_V_10_O_28_ (KNVO)	152.3 mAh g^–1^ at 0.1 A g^–1^ (100% after 1000 cycles at 1 A g^–1^)	^30^
Li_7_[V_15_O_36_(CO_3_)] (Li_7_V1_5_)	135.0 mAh g^–1^ at 3 A g^–1^ (69.5% after 1000 cycles)	^58^
(NH_4_)_2_V_10_O_25_·8H_2_O (MNVO)	162.3 mAh g^–1^ at 20 A g^–1^ (85.6% after 10,000 cycles)	^64^
Na_6_V_10_O_28_ (NVO)	169.5 mAh g^–1^ at 0.1 A g^–1^ (83.79% after 100 cycles)	^65^
KZnV_5_O_14_·2.5H_2_O (KZVO)	186.7 mAh g^–1^ at 1 A g^–1^ (78.7% after 1000 cycles)	^66^
(NH_4_)_8_[V^IV^_12_V^V^_7_O_41_(OH)_9_]·11H_2_O (NVO)	102.2 mAh g^–1^ at 5 A g^–1^ (100% after 88 cycles)	^67^
KPW_12_/SWOH KPW_12_/SW	183 mAh g^–1^ at 5C (84% after 160 cycles) 147 mAh g^–1^ at 5C (68% after 160 cycles)	this work

### 2.3 Charge Storage Mechanism

The ion storage mechanism fundamentally dictates the rate performance and cycling stability of rechargeable batteries. To substantiate this perspective, CV assessments of the nanohybrids were executed at diverse scan rates, varying from 0.1 to 1.2 mV s^–1^. As the scan rate increases, the peaks observed in the CVs systematically broaden, while the overall shape of the CV curves is consistent, as shown in Figure 5a. It can be clearly observed that the CV curves maintained indistinguishable shapes with the oxidation peaks and reduction peaks slightly shifting to higher and lower voltages, respectively. According to eq 1, the power-law formula^57^ has been devised where the peak current (*i*) from the CV profiles relates to the scan rate (*v*) like this:

1 $$i=av^{b}$$

where *a* and *b* are variable parameters.

**Figure 5:**
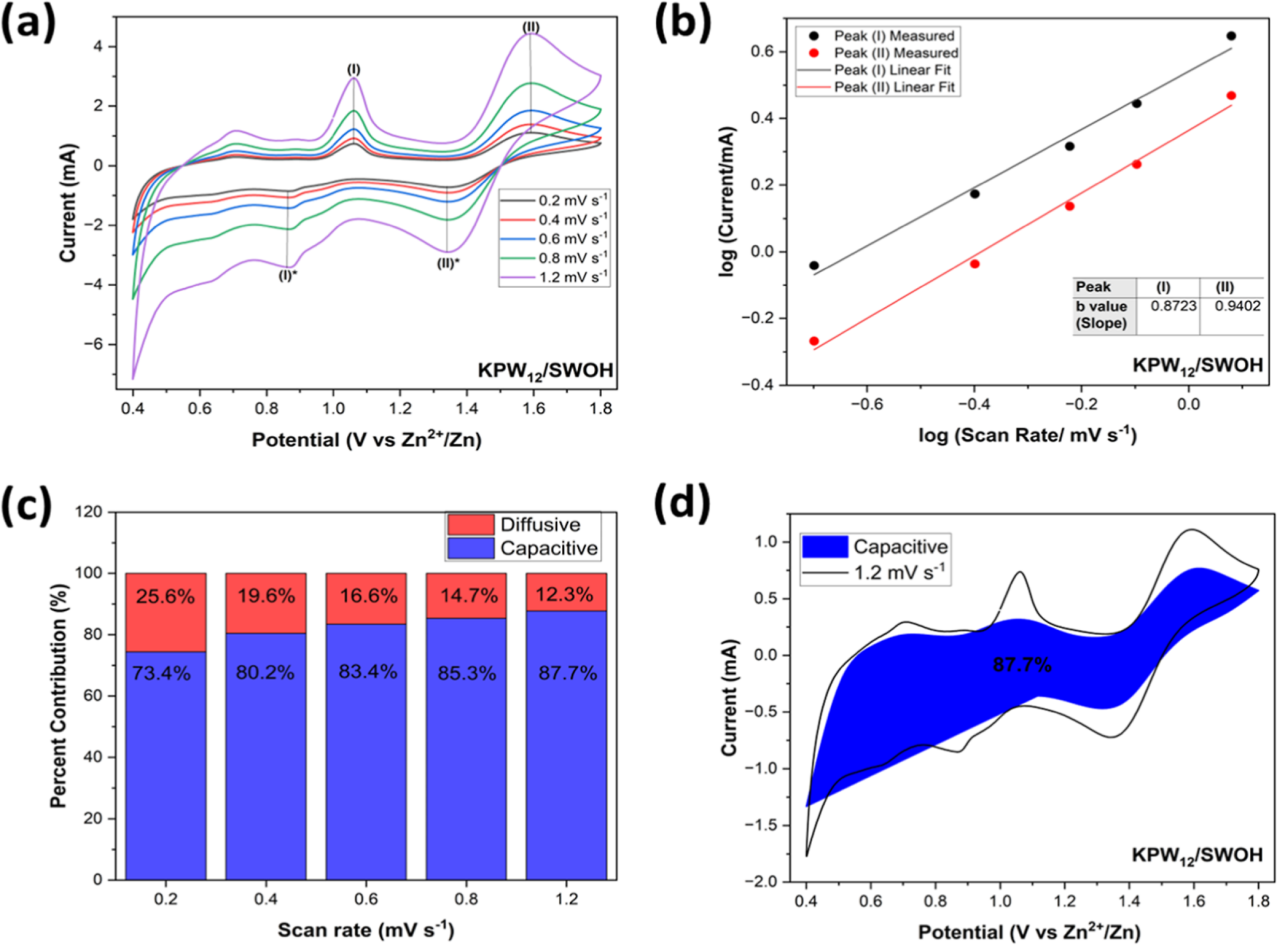
(a) CV curves of the as-synthesized KPW_12_/SWOH nanohybrid at different scan rates. (b) The log current versus log scan rate of anodic peaks. (c) The percentage contribution of capacitive surface-controlled and diffusion-controlled to capacity. (d) The plot of pseudocapacitance contribution as a percentage at a scan rate of 1.2 mV s^–1^.

This can be evaluated with logarithms to give eq 2:

2 log(i)=bxlog(v)+log(a)

The *b* values for the cathode can be calculated as a slope for the plot of log­(*i*) versus log­(*v*). In principle, *b* values approaching 0.5 indicate a diffusion-controlled process (*Q*_s_), while *b* values approaching 1 reveal a surface capacitance-controlled process (*Q*_d_) contribution. As shown in Figure 5b, the deliberated *b* values of the two peaks were calculated to be 0.8723 and 0.9402 for peaks (I) and (II), respectively, indicating that the charge storage mechanism of Zn^2+^ is jointly influenced by both pseudocapacitive and diffusion processes. The pseudocapacitive is, however, dominant, which implies the ability for the KPW_12_/SWOH cathodes to store charge through surface reactions and enhanced multiplicative capacity.^46^ This facilitates rapid Zn-ion diffusion kinetics, contributing to excellent high-rate performance. Dunn’s method was further utilized to investigate the charge storage mechanism.^69^ The capacity is categorized into capacitive surface-controlled (*k*_1_ *v*) and diffusion-controlled (*k*_2_ *v* ^1/2^) parts, as shown in eq 3.

3 $$i\left(v\right)=kk_{1}v+k_{2}v^{1/2}$$

The constants *k*_1_ and *k*_2_ values can be calculated by plotting *i*/*v* ^1/2^ vs *v* ^1/2^ as slopes and the *y*-intercept, respectively (Figure S7). At the different scan rates, the capacitive and diffusion-controlled contributions were calculated, as shown in Figure 5c. With the scan rates of 0.2, 0.4, 0.6, 0.8, and 1.2 mV s^–1^, the corresponding capacitive contributions were 73.4, 80.2, 83.4, 85.5, and 87.7%, respectively. The capacitance contribution significantly exceeds that of the diffusion contribution. At higher scan rates, electrochemical behavior is predominantly influenced by a pseudocapacitive mechanism, which is advantageous for achieving excellent rate performance.^70^ This entails an elevation in the capacitive contribution as both discharge and charge rates escalate.

The capacitance contribution at 1.2 mV s^–1^, 85.3% of Zn^2+^ storage capacity, was associated with the capacitance contribution, as displayed in Figure 5d. The steadily expanding pseudocapacitive contribution verified the pseudocapacitive behavior as the scan rate increased. This behavior gave strong play to the Zn^2+^ storage and release by the nanohybrid and increased the reactive kinetics during the cycle process. The dominance of pseudocapacitance behavior coupled with outstanding electrochemical kinetics leads to superior specific discharge capacity and good Zn^2+^ diffusion coefficients of the KPW_12_/SWOH cathode. Meanwhile, the KPW_12_/SW nanohybrid illustrated a lower ion storage with pseudocapacitance-dominated behavior of 69.8% at 1.2 mV s^–1^ (Figure S8a–d).

### 2.4 Mechanism Analysis

To elucidate further the Zn^2+^ storage mechanism during intercalation/deintercalation, ex situ XRD and SEM/EDX analyses were conducted on the KPW_12_/SWOH cathode under different voltage states. The structural change of the cathode, the GCD curve in Figure 6a, was measured after five discharge–charge cycles, as in the ex situ XRD patterns displayed in Figure 6b, with associated dynamic phase transitions at various charge and discharge stages. The characteristic peaks for PW_12_ are well preserved at the fully charged–discharged cycles, indicating the reversibility in the Zn^2+^ insertion mechanism. However, it is worth noting that during the discharge process these characteristic peaks slightly shifted to a higher angle region and recovered to the pristine stages during the next phase of charging, as demonstrated by the peaks at 31.4° and 36.3° in Figure 6c. This suggested excellent reversibility of Zn^2+^ intercalation/deintercalation into the KPW_12_/SWOH cathode.^71^ Additionally, when discharging at 0.6 V, new notable diffraction peaks emerge at approximately 8.15°, 13.14°, and 18.94° corresponding to the (102), (111), and (103) planes, indexed to the new crystal phase of zinc hydroxy sulfate, Zn_4_SO_4_ (OH)_6_·3H_2_O (ZHS, JPCDS No. 39-0688), that formed as byproduct at the cathodes as reported in previous studies.^72,73^ As the cell charges up to 1.2 V, the intensity of the ZHS characteristic peaks progressively decreases, and this structural transformation is fully reproduced in the subsequent cycles. The reversibility of the behavior was further validated by ex situ SEM/EDX analysis, as shown in Figure 6d,e. In the SEM images, microflake-like ZHS appeared to form on the cathode during the complete discharge process and diminish during the charging phase, signifying the favorable reversibility of the ZHS phase. The EDX analysis during the discharging state shows the uniform distribution of the elements Zn and S, which confirms ZHS, unlike in the fully charged state, where they partly disappeared. These SEM images show that the KPW_12_/SWOH cathode maintains a similar morphology at the charging and discharging states. The preserved morphology after cycling indicates that the cathode experienced no pulverization or structural degradation, thereby contributing to its structural stability and excellent rate performance.

**Figure 6:**
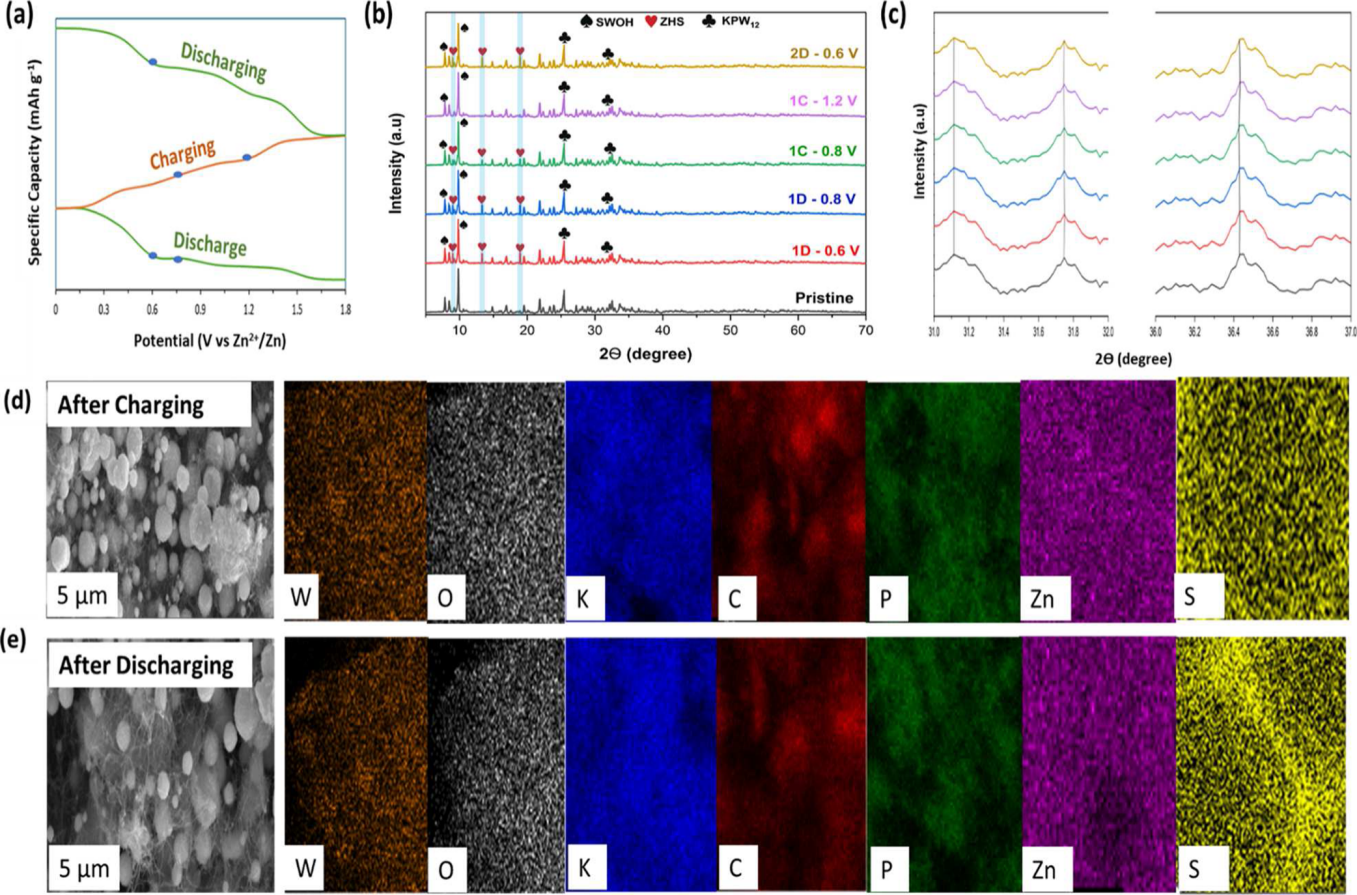
(a) GCD curve for ex situ analysis. (b,c) Ex situ XRD patterns of the KPW_12_/SWOH cathode at the pristine, charged, and discharged states, indicating the appearance of ZHS peaks. The SEM and EDS mapping at 5 μm after (d) charging (e) discharging.

The formation of ZHS is attributed to the side reaction occurring between the dissolved oxygen and the electrolyte, suggesting that H^+^ ions are also involved in the electrochemical reaction, thereby contributing to the specific capacity.^72^ The H^+^ cointercalation with Zn-ions was further confirmed by assembling a cell with 1 M H_2_SO_4_ electrolyte solution, which acted like a proton source from ZnSO_4_ electrolyte, in which KPW_12_/SWOH and zinc foil were used as cathode and anode, respectively.^74^ The CV analysis shown in Figure S9 at 1.2 mV s^–1^ revealed overlapping of peaks, linked to the insertion of pure Zn^2+^ and H^+^ from 1 M ZnSO_4_ electrolyte solution and the 3H^+^ insertion from 1 M H_2_SO_4_/H_2_O electrolyte solution at the potential of 1.03 V. However, observing the enclosed CV curve areas, Zn^2+^ dominates the charge storage process over the 9% capacity contribution of H^+^, partially contributing to the electrochemical performance. Therefore, based on the above results, the electrochemistry involved the cointercalation of Zn^2+^/H^+^ ions into the PW_12_ framework, maintaining structural stability throughout the cycling process.

Drawing from the above discussion, the electrochemical reactions governing the reversible discharge–charge process can be outlined as follows:

anode:

$$\mathrm{Zn}⇌{\mathrm{Zn}}^{2+}+2e^{-}$$

cathode:

$$2KPW_{12}+x{\mathrm{Zn}}^{2+}+\left(x+2\right)e^{-}⇌{\mathrm{Zn}}_{x}{\left(PW_{12}\right)}_{2}+2K^{+}$$

electrolyte:

$$H_{2}O⇌H^{+}+{OH}^{-}$$

$$4{\mathrm{Zn}}^{2+}+6{OH}^{-}+{SO_{4}}^{2-}+3H_{2}O⇌{\mathrm{Zn}}_{4}SO_{4}{\left(OH\right)}_{6}\cdot 3H_{2}O$$

To better comprehend the underlying reaction mechanism, density functional theory (DFT) simulations were conducted by employing the Vienna Ab initio Simulation Package (VASP).^75^ The detailed procedure is listed in Supporting Information. The DFT was first introduced for theoretical analyses and prediction of electron structure and Zn-ion storage properties of the nanohybrids using the density of states (DOS) to fully understand the insertion sites and diffusion paths of Zn^2+^ and H^+^. The DOS analysis, as shown in Figure 7a–c, revealed that nanohybridization significantly reduced the electronic band gaps of 0.15 and 0.4 eV for KPW_12_/SWOH 0 and KPW_12_/SW, respectively, suggesting an improved electronic conductivity of KPW_12_ upon anchoring with SW or SWOH.^76^ Additionally, the H^+^ and Zn^2+^ adsorption energies were compared based on DFT calculation, as shown in Figure 7d. As expected, the KPW_12_/SWOH nanohybrid gave a lower adsorption energy for both H^+^ (−3.69 eV) and Zn^2+^ (−2.43 eV), indicating that they are more easily adsorbed for electrochemical reactions.^73^ The synergistic effect between the redox-active KPW_12_ and the conductive CNT facilitates a lowered adsorption of the ions, making it more feasible for rapid diffusion of H^+^ and Zn^2+^ during the charging/discharging process. This is also in agreement with the energy barrier calculated based on the optimized crystal structures shown in Figure 7e, with KPW_12_/SWOH (0.027 eV) having a lower energy barrier than KPW_12_/SW (0.051 eV) and KPW_12_ (0.12 eV). The DFT calculations revealed that H^+^/Zn^2+^ ions cointercalate into the KPW_12_, with negative insertion energies, indicating the feasibility of the mechanism. Moreover, this strongly suggests that nanohybridization shifts the Fermi level and conduction band, leading to the formation of a distinct intermediate energy level close to the Fermi level and a reduction in the band gap. This reduction in the energy barrier demonstrates that the KPW_12_/SWOH cathode provides an effective Zn-ion migration barrier and enhanced diffusion. As shown in Figure 7f, the active sites for PW_12_ have oxygen in the red regions, which indicates electron density enrichment and accumulation. These regions are mainly distributed between C and O in SWOH, as well as between the terminal W and the terminal O of PW_12_.

**Figure 7:**
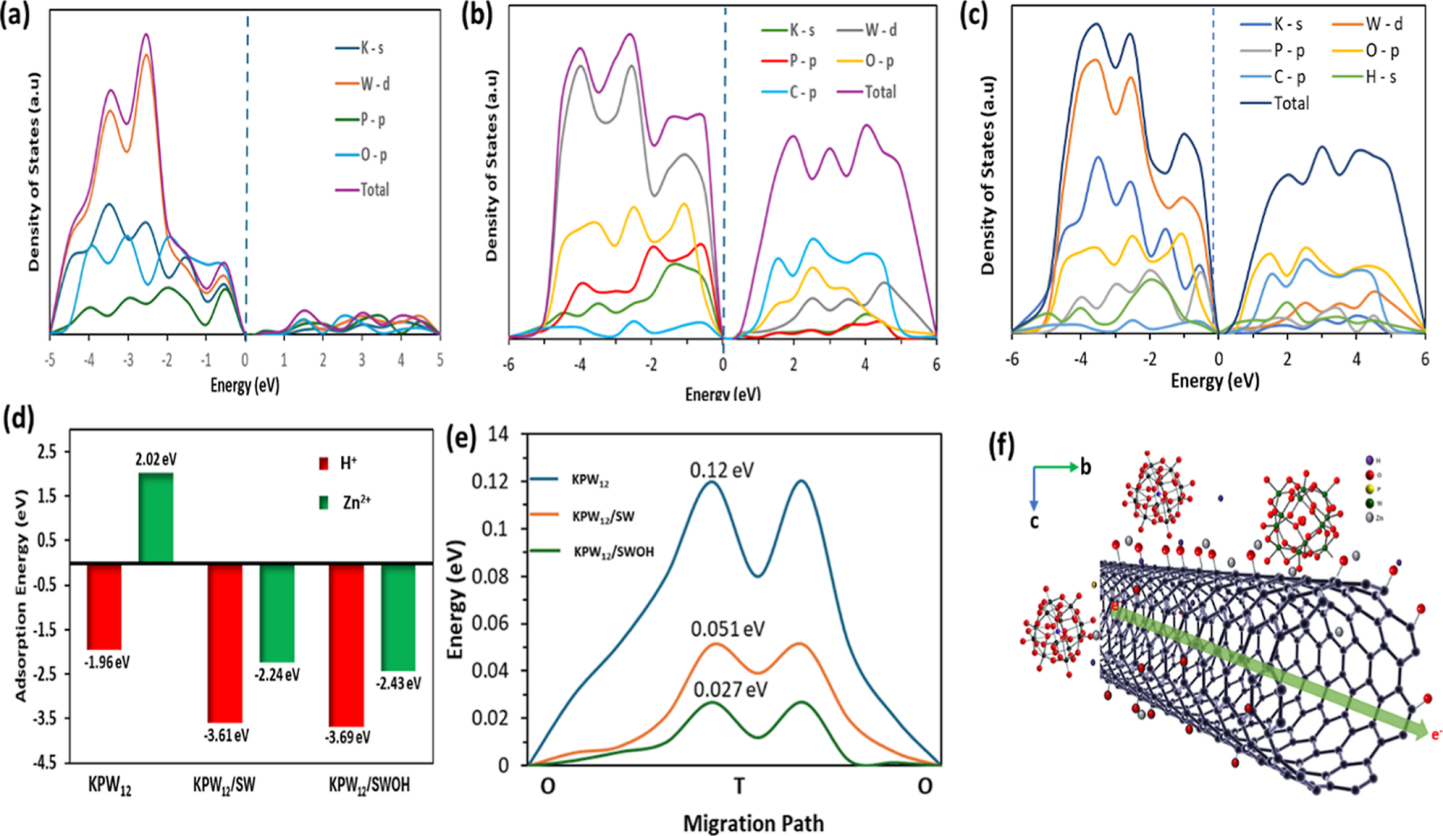
(a–c) The DOS of KPW_12_, KPW_12_/SW, and KPW_12_/SWOH showing changes in band structure upon nanohybridization. (d) The H^+^ and Zn^2+^ adsorption energies of the samples. (e) The energy barrier characteristics of optimized crystal structures. (f) Schematic sketch of the ion and electron movement mechanism of the KPW_12_/SWOH cathode in AZIBs.

In light of the preceding analysis, we can deduce the energy storage mechanism of KPW_12_/SWOH. The maximum of 12 electrons for the Keggin PW_12_ was involved in the redox process, giving a theoretical discharge capacity of 107.8 mAh g^–1^. However, the actual capacity obtained was higher due to the perfect synergistic effect with the SW and SWOH. The notable stability and strong rate performance of the KPW_12_/SWOH cathode can be attributed mainly to its distinctive composition. These theoretical findings are summarized in Figure 8, which illustrates the proposed storage mechanism of this nanohybrid. The Keggin structure accommodates coinsertion of Zn^2+^ and H^+^ without compromising structural stability. The conductive CNT network not only ensures rapid electron transport and structural support but also effectively mitigates the volume changes during cycling. These features collectively enable the performance of the KPW_12_/SWOH cathode in aqueous Zn-ion batteries.

**Figure 8:**
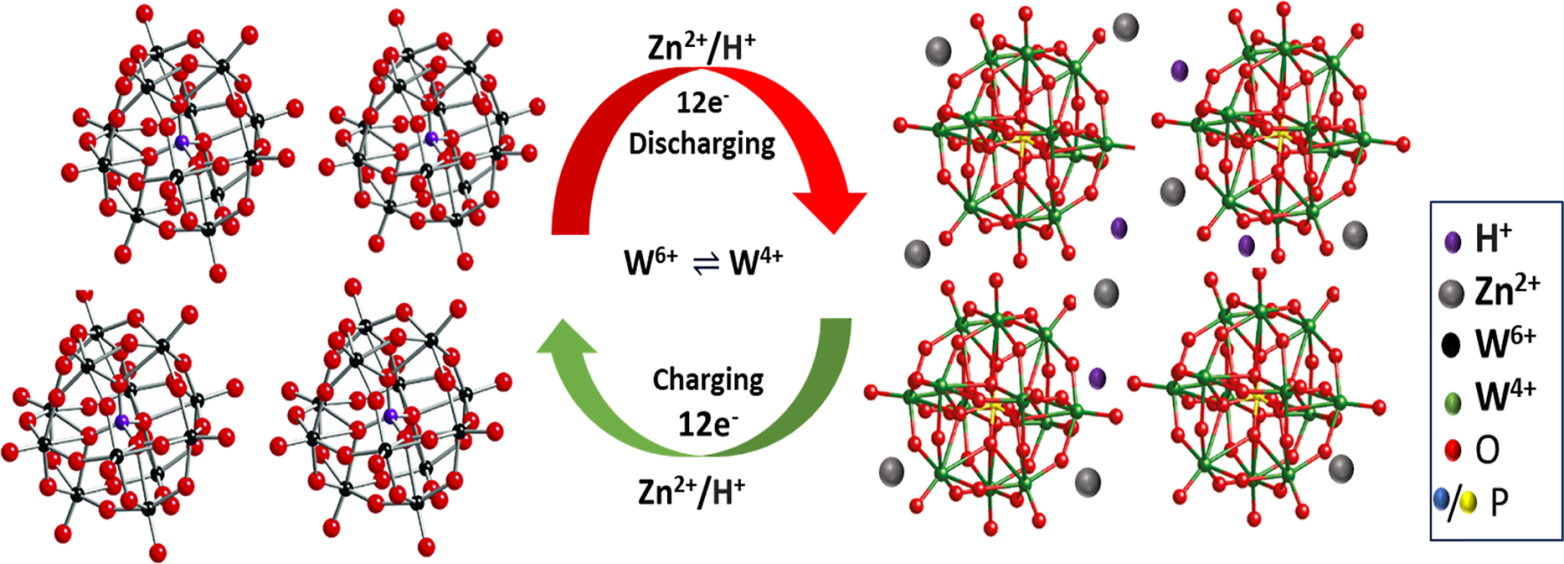
Proposed storage mechanism of Zn-ion for the KPW_12_/SWOH cathode, highlighting ion diffusion, redox activity, and structural features.

## 3 Conclusion

In summary, a facile hydrothermal and ultrasonication procedure was employed in the synthesis of POM/CNT nanohybrids that were investigated for electrochemical properties as a cathode for AZIBs. The resulting KPW_12_/SWOH nanohybrid displayed an outstanding Zn^2+^ storage performance, with an impressive reversible capacity of 183 mAh g^–1^ at the current density of 5C after 160 cycles, with capacity retention of 87%, which is remarkably superior to that of the nanohybrid with unfunctionalized SW and individual KPW_12_ framework. Even at a higher current density of 10C, an excellent discharge capacity of 360.8 mAh g^–1^ was obtained after 50 cycles. The excellent Zn^2+^ storage performance of nanohybrids can be ascribed to the pseudocapacitive nature of the super-reduced state of PW_12_ and the additional active sites provided within the microchannels of SWOH, as well as the perfect synergistic effect brought by the introduction of the electron-conductive SW skeleton. Using the ex situ XRD, SEM/EDX, and DFT calculations, we found that energy storage followed a cointercalation mechanism of both H^+^ and Zn^2+^. This study gives insight and future prospective in the application of POM-based nanohybrids in rechargeable metal ion batteries.

## Supplementary Material



## Notes

SEM images; PXRD pattern of SW and SWOH powder; CV curves of KPW12/SW, SW, and SWOH at the scan rate of 1 mV s-1; UV-vis spectroscopy of electrolytes; GCD curve of KPW12/SW after 50 cycles; Warburg coefficient (σ) calculation; electrochemical performance of non-POM cathode systems for AZIBs; CV curves of the as-synthesized KPW12/SWOH nanohybrid at different scan rates; and CV analysis (PDF)

## †

Department of Pure and Applied Chemistry, School of Natural Sciences, University of Zambia, P.O. Box 32379, Lusaka, 10101, Zambia

## Abbreviations

POM: polyoxometalates
CNT: carbon nanotube
XRD: X-ray diffraction
DFT: density functional theory.
