# Supplementary material for: Investigation on the Keggin Anchored on Hydroxide-Functionalized Single-Walled Carbon Nanotubes as Superior Cathode for Aqueous Zinc-Ion Batteries
Source: ACS Omega. 2025 Aug 5;10(32):36536–49. doi: 10.1021/acsomega.5c05213 (PMC12368648; doi:10.1021/acsomega.5c05213)
Supplement: Supplementary file 1 [file ao5c05213_si_001.pdf]

INVESTIGATION ON THE KEGGIN ANCHORED ON HYDROXIDE FUNCTIONALIZED SINGLE-WALLED CARBON NANOTUBES AS SUPERIOR CATHODE FOR AQUEOUS ZINC-ION BATTERIES.

Langson Chilufya,[†] Vahide Sertbas, Ahmet Aytekin, Engin Karabudak, Mehtap E. Eanes.**

Department of Chemistry, Faculty of Science, Izmir Institute of Technology, Gülbahçe Campus

35430 Urla, İzmir, 35050, Türkiye.

1. Methodology

1.1. Materials

All the materials used in the synthesis were commercially available and purchased, namely phosphotungstic acid ($H_3PW_{12}O_{40}.xH_2O$), potassium chloride (KCl), and acetonitrile were purchased from Aldrich, hydrochloric acid (HCl) 37% from Fisher Chemicals, and methanol from Aga. All the Carbon Nanotubes (CNT), Single Walled (SW)-NK01SW0401 and hydroxide functionalized Single Walled (SWOH)-NK01SW0302, with outside diameter of 1-3nm, length of 5-35 μm, and percent purity of > 65% were purchased from Nanoka Nanotechnology. All the materials were utilized as received without any purification.

1.2. Preparation

1.2.1. Hydrothermal synthesis of mesoporous $K_3PW_{12}O_{40}.xH_2O$ (KPW_{12})

The hydrothermal method was followed in the preparation of KPW_{12}, as reported in the literature.[1] Specifically, 300 mg of KCl was dissolved in 5 mL of H_2O and added dropwise to a solution of 500 mg of $H_3PW_{12}O_{40}.xH_2O$, which was dissolved in 15 mL of H_2O. The resulting precipitate was transferred to a 23 ml PTFE−lined autoclave and placed in an oven at 140 ˚C for 12 hours. Thereafter, it was washed thoroughly with deionized water and oven dried at 90 ˚C for 2 hours.

1.2.2. Synthesis of KPW_{12}/SW nanohybrids

The literature method describing the synthesis of POM/CNT was followed in the preparation of KPW_{12}/SW nanohybrids.[2] Thus, 10 mg of Single Walled CNT (SW) was added to 10%mL of methanol solution and ultrasonically dispersed for 30%minutes at ambient temperature with the sonicator set at four cycles and 60 KHz. Subsequently, KPW_{12} (20%mg) dissolved in 3 mL of Acetonitrile was poured into the dispersed CNT methanol solution. The mixture solution was further ultrasonicated for 2 hours at ambient temperature and the resultant solid was obtained by centrifuging at 5000 rpm for 20 minutes at room temperature. The solution was filtered and washed 3-4 times with water to make sure that all free POMs not attached to SWNT were removed. The final product was dried overnight in a vacuum oven at 60 °C to yield the final black powder product.

For KPW_{12}/SWOH, the same procedure was repeated except that SW was replaced with a hydroxide functionalized SW (SWOH).

1.3. Instrumental characterization

Scanning electron microscopy (SEM) measurements were performed to observe the morphology, size and microstructure of the materials. Powder X-ray diffraction (XRD) patterns were obtained with a Rigaku Smart Lab SE operated at 30 kV and 20 mA with Cu Kα radiation (λ = 0.15406 nm) equipped with Cu-Kα radiation (λ = 0.15418 nm) in the 2θ ranging from 10° to 80° was used to identify the powder phase of the materials. The vibration peaks were obtained using the Thermal Scientific Fourier transform infrared (FT-IR) spectroscopy by mixing the samples with KBr pellets. Thermogravimetric analysis (TGA, STA-449-F3) was conducted in an

air atmosphere, using a heating rate of 10%˚C min^{-1} over a temperature range from 10 to 800%˚C. UV–Vis absorption spectra for all samples were recorded using a Lambda 750 spectrophotometer (PerkinElmer).

1.4. Electrochemical testing

The electrochemical testing of the synthesized nanohybrids was investigated using an electrochemical workstation (Auto Lab, NOVA) using a three-electrode setup. To evaluate the redox activities, the cyclic voltammetry (CV) was performed in a potential range of 0.01 - 3 V *vs.* Zn^{2+}/Zn at different potential sweep rates starting at 0.1 mV s^{-1}. To observe the electrochemical performance of the battery after cyclic reaction, such as rate performance, POM/CNT nanohybrid as active material, conductive carbon black, and PVDF in a mass ratio of 7:2:1, respectively, were mixed in N-methyl-pyrrolidone (NMP), thoroughly ground to make a uniform cathode slurry. This was evenly coated on a Nickel form as the substrate and dried in an oven overnight at 80 ℃. Subsequently, the coated foil was flattened with a roller machine to form the electrodes with a loading amount of approximately 1.0 mg cm^{-2}. With the prepared working electrode as the cathode and metal zinc foil as the anode, a full battery was assembled using commercial glass fiber (Waterman) as the separator and 3.0 M $ZnSO_4$ aqueous solution as the electrolyte. In the co-intercalation analysis, 3.0 M H_2SO_4 and 1.0 M $ZnSO_4$ of electrolytes were used. The galvanostatic charge/discharge (GCD) test was performed using a multichannel battery tester (CT-3008W, NOVA), and all the specific discharge capacities were calculated using the mass of POM composite in the electrode. The electrochemical impedance spectroscopy

(EIS) measurements were performed with a frequency range of 105–10−2 Hz and an amplitude of 5 mV.

Calculation of theoretical capacity, energy density and power density:

The theoretical specific capacity of KPW_{12}, KPW_{12}/SW or KPW_{12}/SW was calculated as outlined in the literature[3], using the following equation:

$$Q_t = \frac{n \times F}{3.6 \times M} \tag{1}$$

Where Q_t, n, F, and M are the theoretical specific capacity, the theoretical number of electrons transferred per molecular structural unit during the redox processes, Faraday's constant (96485 C mol^{-1}), and the molar mass of the samples, respectively. The CNTs mass were not used to calculate the theoretical specific capacity because they did not participate in the redox reaction.[4] Hence, the theoretical specific capacity of KPW_{12}, was 107.8 mAh g^{-1}

The Warburg coefficient, which reflects the inverse relationship with ionic diffusion kinetics, is determined from the slope of the real impedance plotted against the inverse square root of the angular frequency in the low-frequency region.[5] It can be calculated for the electrode using the following equations:

$$R = R_s + R_{CT} + \sigma\omega^{-1/2} \tag{2}$$

Where R denotes the total resistance of the electrodes, R_S is pertains to the resistance values associated with of the electrode and the electrolyte, as derived from the Nyquist plot (Z'' vs Z'), R_{CT} represents the resistance of charge transfer occurring at the interface of the positive electrode and electrolyte obtained at the intercept of the curve with the real axis (Z') at high frequency,

Warburg coefficient (σ), which exhibit an inverse relationship to the $D_{Zn}{}^{2+}$, constitute the slope of the real resistance and reciprocal square root of the angular frequency within the low frequency spectrum.[6]

1.5. Density functional theory

The calculations for Density functional theory (DFT) were conducted by utilizing the Vienna Ab initio Simulation Package (VASP).[7] An energy cut-off set at 400 eV for the plane-wave was used to represent the valence electrons of all elements and expand the wave functions. The interactions between valence electrons and ion cores were described using the projector-augmented wave (PAW) pseudopotential method.[8] The energy convergence criterion between successive steps was set at 1×10^{-5} eV, and during ionic relaxation, the maximum Hellmann-Feynman force acting on each atom is kept below 0.02 eV/Å. All studied compounds are modelled using a $2 \times 1 \times 4$ supercell.[9,10]

2. Supplementary Figures and Tables.

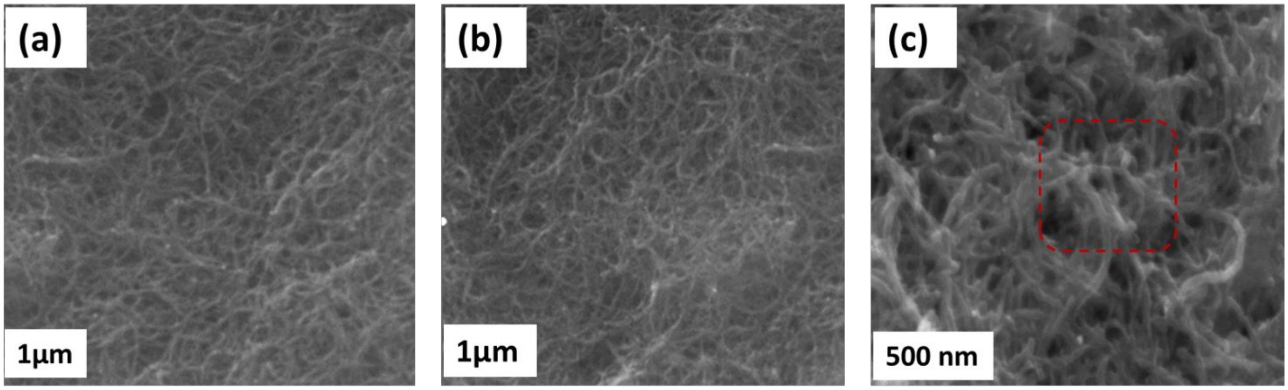

Figure S1. SEM images of (a) SW at 1 µm. (b) SWOH at 1 µm. (c) SWOH at 500 µm.

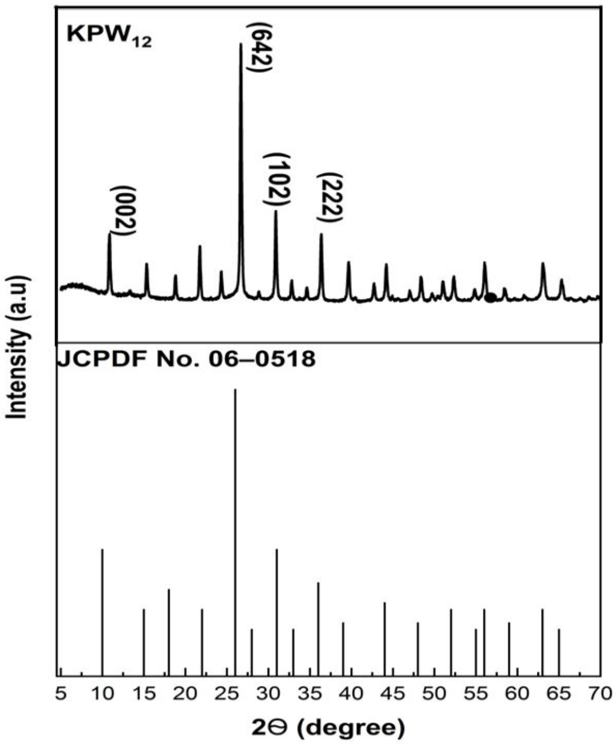

Figure S2. The PXRD pattern of the KPW_{12} crystalline structure matched well with PW_{12} (JCPDF No. 06–0518).

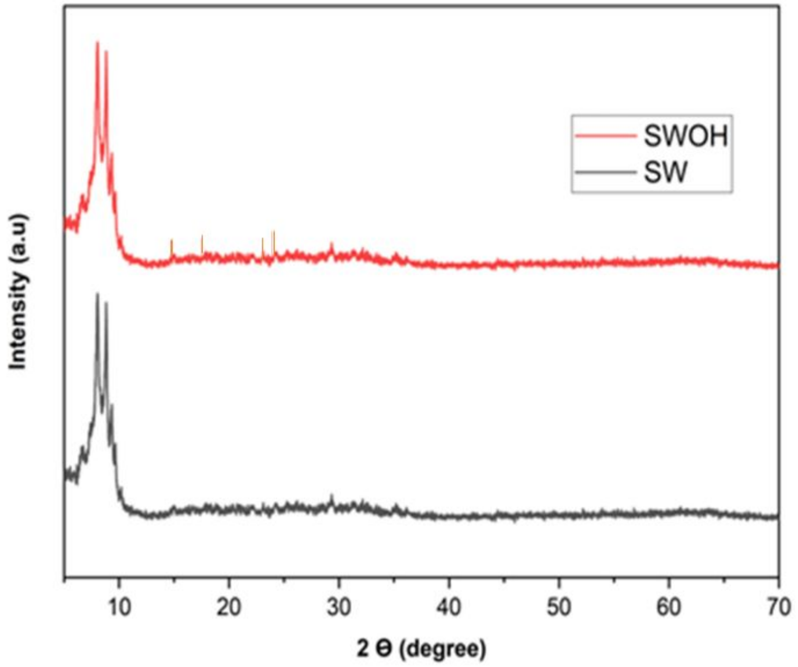

Figure S3. The PXRD pattern of SW and SWOH powder shows their amorphous nature.

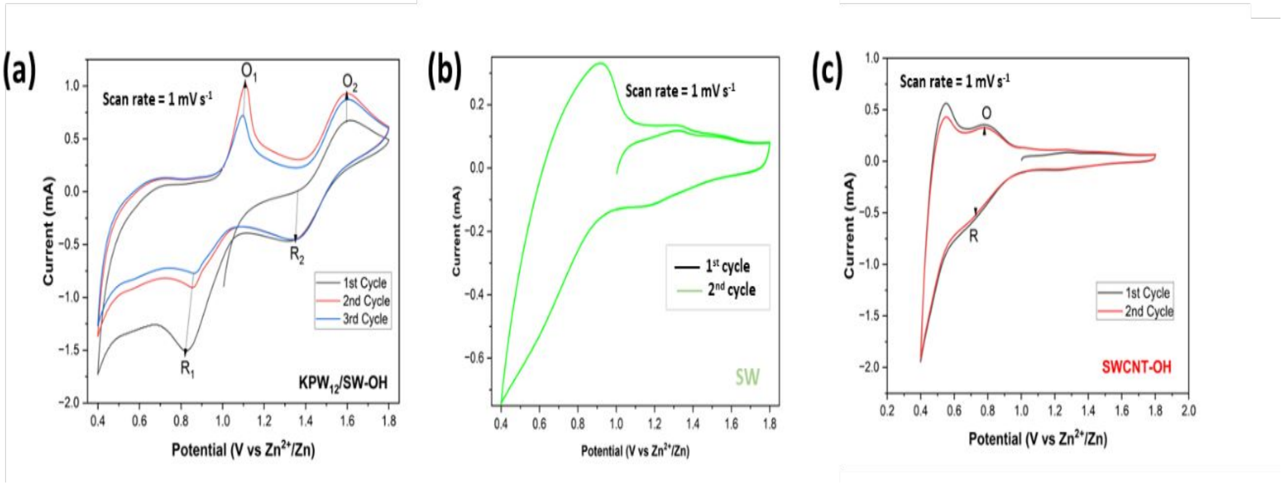

Figure S4. The CV curves of (a) KPW_{12}/SW. (b) SW. (c) SWOH at the scan rate of 1 mV s^{-1}.

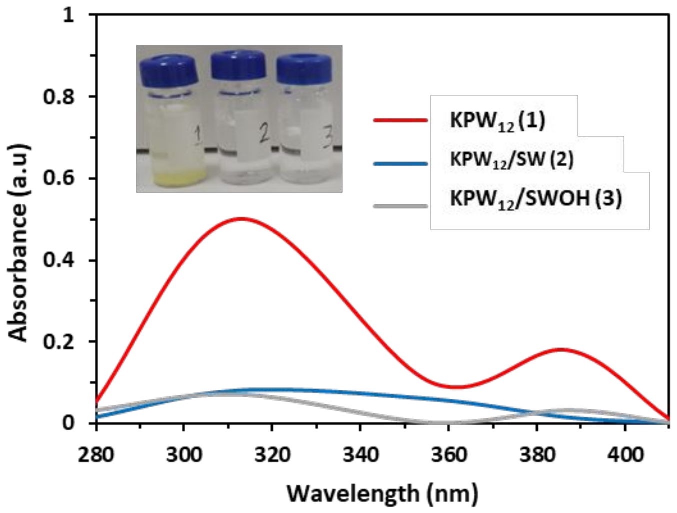

Figure S5. The UV-Vis spectroscopy of electrolytes obtained after five charging/discharge cycles.

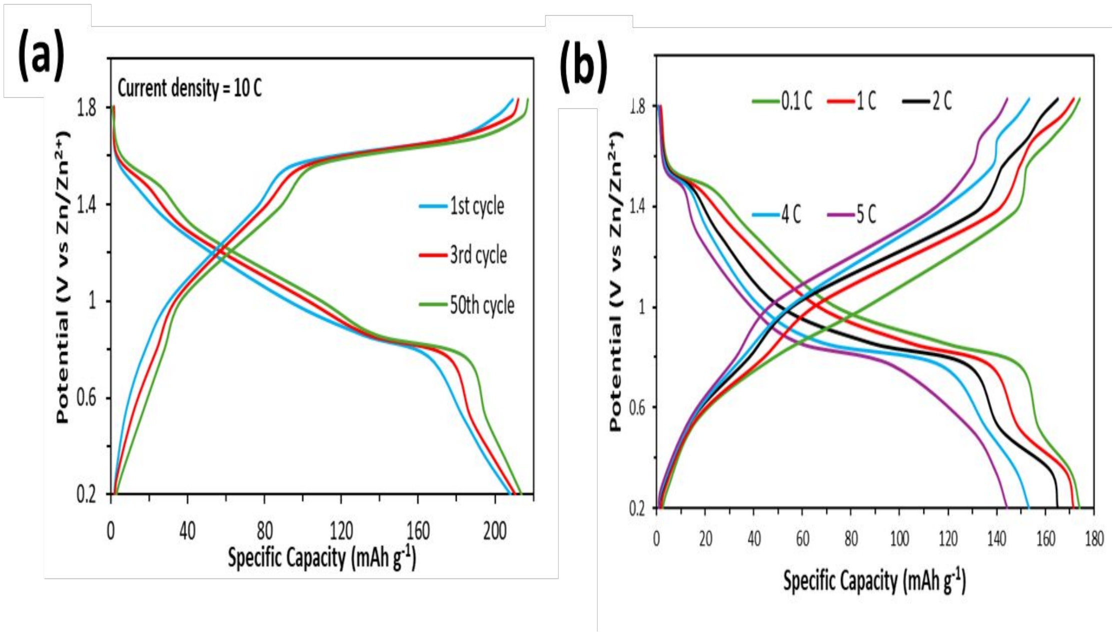

Figure S6. (a) GCD curve of KPW_{12}/SW after 50 cycles at 10 C. (b) GCD curves showing rate performance of KPW_{12}/SW at different current densities.

Table S1. The from Warburg coefficient (σ) calculation

Samples	R_{ct} (Ω)	R_s (Ω)	σ (Ω $S^{-1/2}$)
KPW_{12}	85.05	5.23	91.05
KPW_{12}/SW	53.14	2.38	71.42
KPW_{12}/SWOH	42.54	1.86	38.81

Table S2: The Electrochemical performance of non-POM cathode systems for AZIBs

Type	Cathode materials	Electrochemical performance (Capacity retention, cycle numbers)	Ref.
MOF-Based	Mn-MOF/CNT	260 mAh g^{-1} at 50 mA g^{-1} (80 % after 60 cycles)	11
	Cu-TBPQ MOF	136.7 mAh g^{-1} at 2.0 A g^{-1} (88% after 500 cycles at 1 A g^{-1})	12
Organic Materials	PDBQ	157.39 mAh g^{-1} at 0.1 A g^{-1} (95 % after 100 cycles)	13
	NTCDA-2,3-DNQ,	133.3 mAh g^{-1} at 15 Ag^{-1} (87.1% after 10,000cycles)	14
	[TPA-$(OH)_3$]	123.7 mAh g^{-1} at 5.0 A g^{-1} (95.87% after 200 cycles)	15
	TAHQ	123.7 mAh g^{-1} at 0.5 A g^{-1} (93.7% after 100 cycles)	16
	rPOP	120 mAh g^{-1} at 0.1 A g^{-1} (95 % after 1000 cycles)	17

Hybrid Composites	MnVO	204 mAh g^{-1} at 1.0 A g^{-1} (87% after 50 cycles)	18
	α-MnO_2@ZIF-67	313 mAh g^{-1} at 100 mA g^{-1} (95.2% after 1000 cycles)	19
	CaV_4O_9-MXene	271.6 mAh g^{-1} at 0.1 A g^{-1} (95.2 %, after 50 cycles)	20
	MnSe@rGO	228 mAh g^{-1} at 0.1 C (71.5 %, after 45 cycles)	21
	Fe-MnO/C-3	134 mAh g^{-1} at 1 A g^{-1} (85 %, after 800 cycles)	22
	HVO/GO-CNTs	380 mAh g^{-1} at 0.2 A g^{-1} (82.6 %, after 1000 cycles)	23
	Ca-ddmb@C	314 mAh g^{-1} at 100 mA g^{-1} (89 %, after 179 cycles)	24
	NVO/PoPDA@GO	334 mAh g^{-1} at 1.0 mA g^{-1} (92 %, after 50 cycles)	25
	KPW_{12}/SWOH	183 mAh g^{-1} at 5 C (84 %, after 160 cycles)	This work
	KPW_{12}/SW	147 mAh g^{-1} at 5 C (68 %, after 160 cycles	

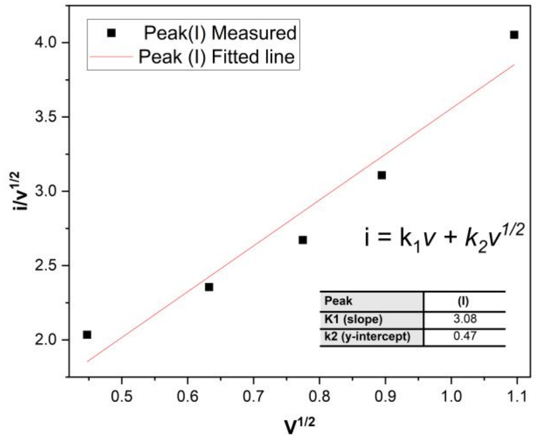

Figure S7. The constants k_1 and k_2 values can be calculated by plotting $i/v^{1/2}$ vs $v^{1/2}$.

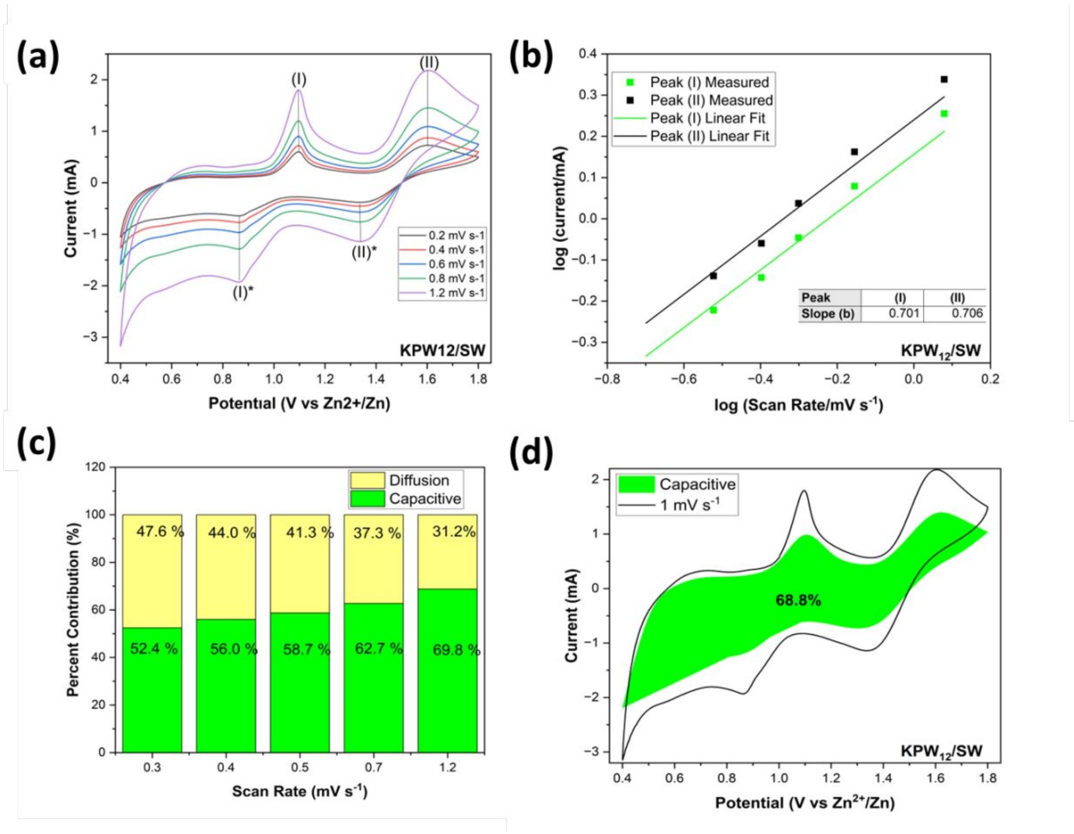

Figure S8. (a) CV curves of the as-synthesized KPW_{12} /SWOH nanohybrid at different scan rates. (b) log current versus log scan rate curves of cathodic and anodic peaks. (c) capacitive

surface-controlled and diffusion-controlled contributions to capacity. (d) The plot of pseudo-capacitance contribution as a percentage at a scan rate of 1.2 mV s⁻¹.

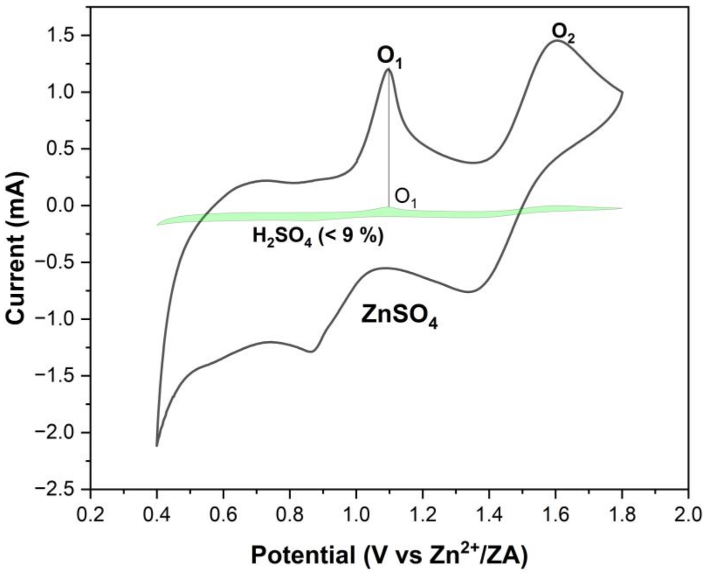

Figure S9. The CV analysis at 0.8 mVs^{-1} associated with pure Zn^{2+} and H^+ insertion from 1 M $ZnSO_4$%electrolyte solution and the 3H^+ insertion from 1 M H_2SO_4/H_2O%electrolyte solution at the potential of 1.03.

AUTHOR INFORMATION

Corresponding Author

Mehtap E. Eanes - Department of Chemistry, Faculty of Science, Izmir Institute of Technology, Gülbahçe Campus 35430 Urla, İzmir, 35050, Türkiye. https://orcid.org/0000-0002-2151-8242. Email: mehtapemirdag@iyte.edu.tr.

Langson Chilufya - Department of Pure and Applied Chemistry, School of Natural Sciences, University of Zambia, P. O. Box 32379, Lusaka, 10101, Zambia. https://orcid.org/0000-0002-3503-7474. Email: langsonchilufya@iyte.edu.tr.

Authors

Authors

Vahide Sertbas - Department of Chemistry, Faculty of Science, Izmir Institute of Technology, Gülbahçe Campus 35430 Urla, İzmir, 35050, Türkiye. https://orcid.org/0009-0005-3941-9698.

Ahmet Aytekin - Department of Chemistry, Faculty of Science, Izmir Institute of Technology, Gülbahçe Campus 35430 Urla, İzmir, 35050, Türkiye. https://orcid.org/0000-0002-2651-5809.

Engin Karabudak - Department of Chemistry, Faculty of Science, Izmir Institute of Technology, Gülbahçe Campus 35430 Urla, İzmir, 35050, Türkiye. https://orcid.org/0000-0002-2948-7102

Present Addresses

†Department of Pure and Applied Chemistry, School of Natural Sciences, University of Zambia, P. O. Box 32379, Lusaka, 10101, Zambia.

ABBREVIATIONS

POM, Polyoxometalates; CNT, Carbon nanotube; XRD, X-ray diffraction; DFT, Density functional theory.

REFERENCES

(1) Li, X.; Xue, H.; Pang, H. Facile Synthesis and Shape Evolution of Well-Defined Phosphotungstic Acid Potassium Nanocrystals as a Highly Efficient Visible-Light-Driven Photocatalyst. *Nanoscale* **2017**, *9* (1), 216–222. https://doi.org/10.1039/c6nr07680g.

(2) Hu, J.; Ji, Y.; Chen, W.; Streb, C.; Song, Y. F. "Wiring" Redox-Active Polyoxometalates To Carbon Nanotubes Using a Sonication-Driven Periodic Functionalization Strategy. *Energy Environ Sci* **2016**, *9* (3), 1095–1101. https://doi.org/10.1039/c5ee03084f.

(3) Li, L.; Liu, S.; Luo, J.; Hou, X.; Kong, J.; Zhang, Q.; Lai, W.; He, C. Three-Dimensional Architecture Design Enables Hexaazatriphenylene-Based Polymers as High-Voltage, Long-Lifespan Cathodes for Aqueous Zinc–Organic Batteries. *eScience* **2025**, 100379. https://doi.org/10.1016/j.esci.2025.100379.

(4) Cao, J.; Xue, Y.; Ji, Z.; Pu, J.; Shen, X.; Kong, L.; Yuan, A. CoNi Hexacyanoferrate Nanoparticles Anchored on Carbon Nanotubes as Superior Cathode Materials for Rechargeable Aqueous Zinc-Ion Batteries. *J Energy Storage* **2024**, *86*. https://doi.org/10.1016/j.est.2024.111413.

(5) Liu, L.; Wang, T.; Zhao, J.; Liu, X.; Cui, J.; Zhang, X.; Sha, J. Insight into Lithiation Mechanism of Co3S4 Anode for Lithium-Ion Batteries Triggered by Keggin POMs and GO. *Chemical Engineering Journal* **2025**, *506*. https://doi.org/10.1016/j.cej.2025.159999.

(6) Hou, Y.; Cui, L.; Wang, Q.; Wang, M.; Li, G.; Yu, K. The Ternary Composite Formed by Intercalation of Porous Keggin-Type POM and Lamellar MoS2 into Accordion-like MXene for Enhanced Electrochemical Properties of the LIBs. *Chemical Engineering Journal* **2025**, *507*. https://doi.org/10.1016/j.cej.2025.160503.

(7) Kresse, G.; Furthmü, J. *Efficient Iterative Schemes for Ab Initio Total-Energy Calculations Using a Plane-Wave Basis Set*; 1996.

(8) Kresse, G.; Joubert, D. *From Ultrasoft Pseudopotentials to the Projector Augmented-Wave Method*.

(9) Jing, F.; Liu, Y.; Shang, Y.; Lv, C.; Xu, L.; Pei, J.; Liu, J.; Chen, G.; Yan, C. Dual Ions Intercalation Drives High-Performance Aqueous Zn-Ion Storage on Birnessite-Type Manganese Oxides Cathode. *Energy Storage Mater* **2022**, *49*, 164–171. https://doi.org/10.1016/j.ensm.2022.04.008.

(10) Grimme, S.; Antony, J.; Ehrlich, S.; Krieg, H. A Consistent and Accurate Ab Initio Parametrization of Density Functional Dispersion Correction (DFT-D) for the 94 Elements H-Pu. *Journal of Chemical Physics* **2010**, *132* (15). https://doi.org/10.1063/1.3382344.

(11) Zhang, J.; Liu, Y.; Wang, T.; Fu, N.; Yang, Z. Manganese-Based MOF Interconnected Carbon Nanotubes as a High-Performance Cathode for Rechargeable Aqueous Zinc-Ion Batteries. *J Energy Storage* **2024**, *76*. https://doi.org/10.1016/j.est.2023.109873.

(12) Liu, J.; Zhou, Y.; Xing, G.; Qi, M.; Tang, Z.; Terasaki, O.; Chen, L. 2D Conductive Metal–Organic Framework with Anthraquinone Built-In Active Sites as Cathode for Aqueous Zinc Ion Battery. *Adv Funct Mater* **2024**, *34* (21). https://doi.org/10.1002/adfm.202312636.

(13) Sajid, M.; Ur Rahman, S.; Tao, F.; Xie, D.; Ur Rehman, M. S.; Salah, A.; Zhang, J. Highly Stable Cyclic Performance of Benzoquinone-Based Organic Cathode in Aqueous Zinc Ion Batteries. *Batter Supercaps* **2025**, *8* (6). https://doi.org/10.1002/batt.202400675.

(14) Wang, J.; Gao, X.; Wang, Y.; Pan, R.; Liu, Z.; Liu, X.; Xie, H.; Yu, F.; Wang, G.; Gu, T. Robust Ring Insoluble Naphthoquinone Derivative Cathode with High Loading and Long Cycle Life for Aqueous Zinc Organic Batteries. *Nano Research Energy* **2024**. https://doi.org/10.26599/nre.2024.9120124.

(15) Huang, J.; Li, M.; Li, Z.; Han, C.; Li, Y. Stable Triphenolamine Radical Cathode with High Electron Conductivity for High-Rate Aqueous Zinc-Ion Batteries. *ACS Appl Mater Interfaces* **2025**. https://doi.org/10.1021/acsami.5c01552.

(16) Wang, Y.; Niu, S.; Gong, S.; Ju, N.; Jiang, T.; Wang, Y.; Zhang, X.; Sun, Q.; Sun, H. bin. Fused Functional Organic Material with the Alternating Conjugation of Quinone–Pyrazine as Cathode for Aqueous Zinc Ion Batteries. *Small Methods* **2024**, *8* (7). https://doi.org/10.1002/smtd.202301301.

(17) Buyukcakir, O.; Yuksel, R.; Begar, F.; Erdogmus, M.; Arsakay, M.; Lee, S. H.; Kim, S. O.; Ruoff, R. S. Ultralong-Life Quinone-Based Porous Organic Polymer Cathode for High-Performance Aqueous Zinc-Ion Batteries. *ACS Appl Energy Mater* **2023**, *6* (14), 7672–7680. https://doi.org/10.1021/acsaem.3c01163.

(18) Narsimulu, D.; Krishna, B. N. V.; Shanthappa, R.; Bandi, H.; Yu, J. S. High-Capacity and Long-Life Manganese Vanadium Oxide Composite as a Cathode for Aqueous Zinc-Ion Batteries. *Adv Mater Technol* **2023**, *8* (18). https://doi.org/10.1002/admt.202300484.

(19) Bai, M.; Li, S.; Zhang, C.; Liu, Y.; Wen, Z.; Sun, J. Novel Composite Cathode Material α-MnO2@ZIF-67 for High Performance Aqueous Rechargeable Zn-Ion Battery. *Journal of Electroanalytical Chemistry* **2024**, *957*. https://doi.org/10.1016/j.jelechem.2024.118104.

(20) Fang, L.; Lin, L.; Wu, Z.; Xu, T.; Wang, X.; Chang, L.; Nie, P. High-Performance Layered CaV4O9-MXene Composite Cathodes for Aqueous Zinc Ion Batteries. *Nanomaterials* **2023**, *13* (9). https://doi.org/10.3390/nano13091536.

(21) Feng, Y.; Yao, Y.; Wang, S.; Ma, X.; Han, Y.; Feng, J.; Wen, J.; Tian, R.; Sun, Q.; Tian, Y. Role of Hetero-Doped Reduced Graphene Oxide in Suppressing Elemental Dissolution in Manganese Selenide Cathode for Aqueous Zinc-Ion Batteries. *ChemSusChem* **2024**. https://doi.org/10.1002/cssc.202402101.

(22) Pan, G.; Wang, Z.; Zhang, J.; Cao, M.; Zhang, L.; Zhang, J. Porous Carbon-Coated Fe-Doped MnO as High-Performance Cathode for Aqueous Zinc Ion Batteries. *Energy Technology* **2024**. https://doi.org/10.1002/ente.202401690.

(23) Rao, D.; Zhang, W.; Cheng, B.; Wang, Y.; Lei, C.; An, Q.; Huang, M.; Mai, L. V2O5 · NH2O and Graphene Oxide/CNTs Composite Film as Binder-Free Cathode for Aqueous Zinc-Ion Batteries. *Batter Supercaps* **2024**, *7* (5). https://doi.org/10.1002/batt.202400046.

(24) Zhu, M.; Gao, Q.; Zheng, M.; Wang, L.; Sun, Y.; Liu, J.; Wu, S. Designing a 2D Calcium Coordination Polymer Interconnected with Carbon as a High-Performance Cathode for Aqueous Zinc-Ion Batteries. *ACS Sustain Chem Eng* **2025**. https://doi.org/10.1021/acssuschemeng.4c06725.

(25) Li, M.; Liu, M.; Lu, Y.; Zhang, G.; Zhang, Y.; Li, Z.; Xu, Q.; Liu, H.; Wang, Y. A Dual Active Site Organic–Inorganic Poly(O-Phenylenediamine)/NH4V3O8 Composite Cathode Material for Aqueous Zinc-Ion Batteries. *Adv Funct Mater* **2024**, *34* (19). https://doi.org/10.1002/adfm.202312789.
